# Integrating physiology, genetics, and transcriptome to decipher a new thermo-sensitive and light-sensitive virescent leaf gene mutant in cucumber

**DOI:** 10.3389/fpls.2022.972620

**Published:** 2022-08-16

**Authors:** Zhipeng Zhang, Jinyao Wang, Guoming Xing, Meilan Li, Sen Li

**Affiliations:** ^1^College of Horticulture, Shanxi Agricultural University, Jinzhong, China; ^2^Collaborative Innovation Center for Improving Quality and Increase of Protected Vegetables in Shanxi Province, Jinzhong, China

**Keywords:** cucumber, virescent leaf, male-sterile, fine mapping, BSA-Seq, RNA-Seq, RST1

## Abstract

Chloroplasts are the material basis of photosynthesis, and temperature and light severely affect chloroplast development and thus influence photosynthetic efficiency. This study identified a spontaneous virescent leaf mutant, SC311Y, whose cotyledons and true leaves were yellow and gradually turned green. However, temperature and light affected the process of turning green. In addition, this mutant (except at the seedling stage) had ruffled leaves with white stripes, sterile males, and poorly fertile female flowers. Genetic characteristics analysis revealed that the recessive gene controlled the virescent leaf. Two F_2_ populations mapped *v-3* to the interval of 33.54–35.66 Mb on chromosome 3. In this interval, BSA-Seq, RNA-Seq, and cDNA sequence analyses revealed only one nonsynonymous mutation in the *Csa3G042730* gene, which encoded the RNA exosome supercomplex subunit resurrection1 (RST1). *Csa3G042730* was predicted to be the candidate gene controlling the virescent leaf, and the candidate gene may regulate chloroplast development by regulating *plastid division2 (PDV2)*. A transcriptome analysis showed that different factors caused the reduced chlorophyll and carotenoid content in the mutants. To our knowledge, this study is the first report of map-based cloning related to virescent leaf, male-sterile, and chloroplast RNA regulation in cucumber. The results could accelerate the study of the RNA exosome supercomplex for the dynamic regulation of chloroplast RNA.

## Introduction

Green leaves are the material basis of plant photosynthesis and the source of plant energy. Thus, chlorophyll synthesis abnormalities, anthocyanin metabolism abnormalities, and chloroplast development decomposition negatively changed the leaf color (Zhao et al., 2020). Virescent leaf mutations represent a significant portion of leaf color mutations in various plants. The cotyledons and true leaves of virescent leaf mutants are initially white, yellow, or light green and gradually turn to normal green as the plant grows, but most plants are thermo- or light-sensitive. The first virescent leaf mutants were identified in maize (*Zea mays*; Emerson, 1912; Demerec, 1924) and confirmed as thermo-sensitive (Phinney and Kay, 1954). The virescent leaf mutant in bean (*Phaseolus vulgaris*) is light-sensitive (Dale and Heyes, 1970). In addition, a light-sensitive maternally inherited virescent leaf mutant was identified in tobacco (*Nicotiana tabacum* and *N. suaveolens*; Archer and Bonnett, 1987). Another light-sensitive virescent leaf mutant was identified in Arabidopsis (Brusslan and Tobin, 1995). However, the virescent leaf mutant in rice (*Oryza sativa*) is temperature-sensitive (Sugimoto et al., 2007). Virescent leaf mutants are also found in other plants, including barley (*Hordeum vulgare*; Maclachlan and Zalik, 1963), cotton (*Gossypium hirsutum*; Benedict and Ketring, 1972), peanut (*Arachis hypogaea*; Benedict and Ketring, 1972), and maize (*Zea mays*; Langdale et al., 1987).

Mutants of the Arabidopsis *resurrection1* (*rst1*) gene are characterized by an abnormal waxy epidermis, abnormal leaf shape, late inflorescence emergence, and stunted embryo development, and they produce nonviable seeds (Chen et al., 2005). *RST1* plays a role in plant defense (Mang et al., 2009) and controls the dynamic balance of plant membrane proteins (Zhao et al., 2019). Recent research on *RST1* has made breakthroughs (Lange et al., 2019; Li et al., 2019; Auth et al., 2021). For example, the formation of higher-order complexes by RNA exosomes and RST1 has been identified that catalyze RNA maturation and degradation (Lange and Gagliardi, 2022; Lee and Suh, 2022). Moreover, mutations in higher-order complexes cause several leaf color mutations (Sauret-Güeto et al., 2006; Kobayashi et al., 2007; Marchive et al., 2009).

Cucumber, *Cucumis sativus L*. (2*n* = 2*x* = 14), is a popular vegetable that ranks third globally in production and sixth in the area grown in 2020 [Food and Agriculture Organization (FAO), accessed on May 1, 2022]. Cucumber has many leaf color mutants, including *chlorophyll deficient (cd)*, *light-sensitive (ls)*, *pale lethal (pl)*, *golden leaf (g)*, *golden cotyledon (gc)*, *yellow cotyledons-1 (yc-1)*, *yellow cotyledons-2 (yc-2)*, *yellow plant (yp)*, *yellow stem (ys)*, *light green cotyledons-1 (lg-1)*, *light green cotyledons-2 (lg-2)*, *virescent (v)*, and *variegated virescent* (*vvi*; Pierce and Wehner, 1990). Others include *virescent-1* (*v-1*; Miao et al., 2016), *virescent-yellow leaf* (*vyl*; Song et al., 2018), *variegated leaf* (*vl*; Cao et al., 2018), *yellow-green leaf* (*yg1*; Ding et al., 2019), *virescent-2* (*v-2*; Zhang et al., 2020), *yellow young leaf-1* (*yyl-1*; Hu et al., 2020), *yellow leaf 2.1* (*yl2.1*; Xiong et al., 2020), and *albino* (Yan et al., 2020).

Some map-based cloned mutants, such as the virescent cotyledon and virescent true leaf mutant *v-1*, are presumably caused by mutations in the *CsaCNGCs*-encoding cyclic-nucleotide-gated ion channel proteins (Miao et al., 2016). In mutant *yp*, the whole plant is golden, possibly due to a mutation in the magnesium chelatase I subunit of the chlorophyll biosynthetic pathway (Gao et al., 2016). However, EMS mutagenesis generated the *vl* mutant in cucumber, presumably due to a mutation that encodes chorismate synthase (Cao et al., 2018). EMS mutagenesis also resulted in the photosensitive yellow leaf mutant, *vyl*, presumably due to a mutation that encodes a DnaJ-like zinc finger protein (Song et al., 2018). In addition, the spontaneous mutant *yg1* with yellow-green leaves is probably caused by mutations in the *tandem 13-lipoxygnease (LOX)* genes (Ding et al., 2019). The virescent leaf spontaneous mutant *v-2* could possibly be caused by a mutation in *Csa3G890020*, which encodes the auxin F-box protein (Zhang et al., 2020). An EMS mutagenesis in the CsHD that contains the HD (histidine and aspartate) structural domain proteins synthesizes the yellow leaf mutant, *yyl-1*. In the EMS mutagenized population, cotyledons and true leaves start yellow before slowly turning green (Hu et al., 2020). Another population mutagenized by EMS generated the yellow leaf mutant *yyl2.1*, with yellow cotyledons. The first true leaf is light green; the second is dark yellow, and the third does not turn green, possibly due to a mutation in *Csa2G263900*, which encodes pdTPI (Xiong et al., 2020).

In this study, a new thermo-sensitive and light-sensitive virescent leaf mutant SC311Y was identified by spontaneous mutation. The cotyledons and newly grown true leaves of this mutant were yellow and gradually turned green. This study described the location of candidate genes that encode the RST1 protein, transcriptomic differences, phenotypic, physiological, and genetic characteristics in cucumber. These findings will accelerate studies of the RNA exosome super complex for the dynamic regulation of chloroplast RNA.

## Materials and methods

### Plant material and phenotype data collection

The virescent leaf (*v-3*) mutant is a spontaneous mutant isolated from SC311 (PI 164465), an Indian-derived selfing line that originated from the USDA, with normal green cotyledons and true leaves that produce normal flowers and fruits. SC311Y was found when the fields were tended. Variety “9930” (also known as “Chinese long”) is a north China fresh market-type cucumber with normal green cotyledons and true leaves. Variety “9930” was sequenced in 2009 and is often used as a hybrid plant (Huang et al., 2009). In this study, the F_2_-A population was constructed by self-crossing heterozygous SC311 and the F_2_-B population, which was constructed using *v-3* (female) and “9930” (male). All the F_2_ populations were grown in an artificial climate chamber in the experimental building of Shanxi Agricultural University (Shanxi, China). The plants were transplanted to the greenhouse at two true-leaf stage. The photoperiod of the artificial climate chamber was 16/8 h; the temperature was 25/18°C, and the light was 25000/0 Lx. The color of cotyledons and true leaves was determined by the naked eye.

### Determining the chlorophyll content and leaf pigment composition

When the plants had grown three true leaves, 0.1–0.2 g of these leaves were sampled from five flattened SC311 wild type (WT) and *v-3* mutants as previously described (Gao, 2006). The samples were cut into filaments, placed in 80% acetone, and extracted in a 50 ml centrifuge tube for 1 day. They were shaken twice within the extraction time. The absorbance at 663, 646, and 470 nm was measured using an UV spectrophotometer (UV-2600i, Shimadzu, Kyoto, Japan). The contents of chlorophyll a (Ca), chlorophyll b (Cb), and carotenoids (Cx·c) were calculated according to the following equations (Lichtenthaler, 1987):


$$\mathrm{Ca}=\left(12.21\times \mathrm{OD}663-2.81\times \mathrm{OD}646\right)\times V/\left(1000\times M\right)$$

$$\mathrm{Cb}=\left(20.13\times \mathrm{OD}646-5.03\times \mathrm{OD}663\right)\times V/\left(1000\times M\right)$$

$$\begin{array}{l} \mathrm{Cx}.c=\left(1000\times \mathrm{OD}470-3.27\times \mathrm{Ca}-104\times \mathrm{Cb}\right)\\ \;\;\;\;\;\;\;\;\;\;\;\;\;\;\;\;\;\;\times V/\left(229\times 1000\times M\right) \end{array}$$

where *V* is the total volume of the extraction solution, and M is the weight of the sample.

The extracts were scanned using a UV-2600i UV spectrophotometer at a 300–800 nm wavelength range, and the spectral scans were plotted using Microsoft Excel 2019 (Redmond, WA, United States).

### Transmission electron microscopy

Visually, the color of mutant leaves differed greatly. Thus, the SC311 and *v-3* leaves were subjected to transmission electron microscopy (TEM) when the first true leaves of the plants unfolded. The leaves were first placed in a solution of 3.5% glutaraldehyde, vacuumed to submerge the leaves in the liquid, and fixed for 2 days. Afterward, the leaves were fixed in 1% osmium tetroxide (OsO_4_), dehydrated using an alcohol gradient, and embedded using pure resin. The leaves were sliced into 80–100 nm pieces using a microtome (EM UC7, Leica Biosystems, Wetzlar, Germany), stained with uranyl acetate and lead citrate, and observed under a TEM (H7500, Hitachi, Tokyo, Japan).

### Different light intensity and temperature treatments

Mutant *v-3* responds to temperature and light, but the time and degree of color change from yellow to green were inconsistent under different temperatures and light intensities. High temperatures always accompany strong light in a greenhouse. Thus, an experiment was designed to determine the dominant factors of light and temperature on mutant color change. Three temperatures (21, 28, and 35°C) and light intensities (10,000, 25,000, and 50,000 Lx) treatments were selected considering 28°C and 25,000 Lx as the normal temperature and light intensity, respectively. The artificial climate chamber treatments were 1W28T (light: 10,000 Lx, temperature 28°C), 2W28T (light: 25,000 Lx, temperature 28°C), 5W28T (light: 50,000 Lx, temperature 28°C), 2W21T (light: 25,000 Lx, temperature 21°C), and 2W35T (light: 25,000 Lx, temperature 35°C). The flattening cotyledons were sampled at 0, 2, 4, 6, and 8 days, each including 5–10 uniform-sized plants per treatment. The chlorophyll and carotenoid contents under different treatments were identified, and the mean values were calculated after removing outlier values.

### Measurements of photosynthesis, light response, and chlorophyll fluorescence

Ten uniform-sized plants were selected at the third true leaf stage when the leaf size of the plants was more than 5 cm × 4 cm (due to the size of the sealed opening of the LED probe of the LI-6400 of 4 cm × 3 cm). The photosynthetic parameters included the following: net photosynthetic rate (Pn), stomatal conductance (Gs), intercellular CO_2_ concentration (Ci), and transpiration rate (Tr), which were measured in an artificial climate chamber using a portable LI-6400XT photosynthesis system (LI-COR Biosciences, Lincoln, NE, United States).

Before measuring the light response, the plants were treated under saturated light intensity for 15 min to achieve the maximum leaf photosynthetic efficiency. The light intensities were 1,126, 906, 757, 607, 457, 303, 146, 108, 32, 11, and 0 μmol·m^−2^·s^−1^. The light response curve was measured using the automatic program of the portable photosynthesis system. The Photosyn Assistant V1.0 was then used to fit the photo response curves.

After 20 min of adaptation to darkness, the initial fluorescence (F_o_) was measured using the IMAGING-PAM modulated chlorophyll fluorescence imaging system (Heinz Walz GmbH, Effeltrich, Germany). The maximum fluorescence (F_m_) was measured after a saturation pulse of light. The leaves were irradiated with actinic light for approximately 10 min before subjection to saturated pulse light at a stable real-time fluorescence (F_t_) to measure the maximum fluorescence (F_m_′). The IMAGING-PAM instrument directly calculated photosystem II actual photochemical efficiency Y(II), the quantum yield of regulated energy dissipation Y(NPQ), non-regulated energy dissipation Y(NO), non-photochemical quenching coefficient (NPQ), and photochemical quenching coefficient (qP and qL). The maximum quantum yield of photosystem II is F_v_/F_m_ = (F_m_-F_o_)/F_m_. The photosynthetic measurements and chlorophyll fluorescence parameters were determined using six plants with visibly uniform growth and health that were within the same location.

### The *v-3* mapping strategy

DNA was extracted from the SC311 (WT), “9930” (WT), and *v-3* as previously described (Murray and Thompson, 1980). The simple sequence repeat (SSR) markers of “9930” (Ren et al., 2009) and “Gy14” (Cavagnaro et al., 2010) were considered when selecting SSR markers. The 220 pairs of the selected SSR markers that were evenly distributed on the seven cucumber chromosomes were selected to determine the polymorphisms of the three parents. The bulked segregation analysis (BSA) method (Michelmore et al., 1991) was used to analyze the linkage between markers and mutants. Essentially, seven green-leafed plants were randomly selected from the F_2_-A population to form the green leaf pool (WT) and seven yellow-leafed plants to form the yellow leaf pool (mutant). A total of 77 individuals of the same fruit were selected from the F_2_-A populations and genotyped using the polymorphic markers. Next, the population was expanded to 177 plants after observing no recombination events between the flanking markers. In the F_2_-B population, 93 individuals were randomly selected and genotyped using the polymorphic markers, and the results were used to construct a preliminary genetic map. After observing no recombination events between the flanking markers, the population size was expanded to 3,903 plants from F_2_-B population. All the primers were commercially synthesized (Tsingke Biotechnology, Beijing, China), and the markers are listed in Supplementary Table 1. The linkage map was drawn using Join Map 4.0 with a limit of detection (LOD) threshold score of 4.0.

### BSA-Seq analysis of the *v-3* locus

In the F_2_-A population grown to the trifoliate stage, 15 leaves of extremely green individuals (WT) and 15 leaves of extremely yellow individuals (mutant) were pooled (equal weight) to form the G-pool and Y-pools, respectively. The pooled samples were sequenced using an Illumina NovaSeq 6000 (Illumina, San Diego, CA, United States) using the PE150 sequencing mode.

The raw data were filtered by removing the adapter sequences, reads containing >5% N, and low-quality reads [where low-quality bases (Q ≤ 10) accounted for >20% of the whole read]. The remaining clean reads were mapped to the reference genomes “Gy14” and “9930” using BWA (0.7.12), and the results were processed using SAMtools (1.9). Next, single nucleotide polymorphisms (SNPs) and insertion–deletion polymorphisms (indels) were identified using GATK (4.0.4.0) and SNPeff (4.3) for structural annotation of the mutant loci.

The target region was established using the Euclidean Distance (ED) algorithm. The SNP with genotypically different loci between the two pools was used to count the base depth and calculate the ED value of each locus. The second power of the original ED was considered as the association value for eliminating the background noise. Next, the ED value was fitted using the LOESS local linear regression method, and a sliding window calculation was performed to filter the regions with the top 1% ED values.

### RNA-Seq

When the cotyledons were fully expanded, they were collected from six SC311 and six *v-3* plants. Equal amounts of tissue from three plants (WT or v-3) were pooled to make one sample. The samples were immediately placed in liquid nitrogen. Total RNA was extracted using a mirVana™ miRNA Isolation Kit (Ambion, Inc., Austin, TX, United States), following the manufacturer’s instructions. Next, cDNA libraries were synthesized using the TruSeq Stranded mRNA LT Sample Prep Kit (Illumina) according to the manufacturer’s instructions. Finally, the constructed libraries were sequenced using an Illumina HiSeq™ 2500 to generate PE150 data.

The clean data were obtained using Trimmomatic. The clean reads were then mapped to the “ChiniseLong _V3” and “Gy14 _V2” genomic sequences using hisat2. The gene counts per sample were obtained using htseq-count. The counted reads were transformed to fragments per kilobase per million mapped reads (FPKM). Differentially expressed genes (DEGs) were identified using the DESeq R package with the estimateSizeFactors and nbinomTest functions. Finally, the DEGs were selected based on the threshold of *q*-value < 0.05 and fold change > 1.5 as the cut-off for identification. Next, Gene Ontology (GO) and Kyoto Encyclopedia of Genes and Genomes (KEGG) enrichment analyses were performed on the DEGs to study the effects of mutants on the biological functions or pathways of the plants.

### Gene prediction and candidate gene identification

The number and function of genes in the target region were predicted from the genomic data (ChiniseLong_V3 and Gy14 _V2) in the Cucurbita genome database.^1^ The DEGs were taken as intersections of the mutations (nonsynonymous, spliced position, and in-frame mutations). Specific primers were designed to clone and sequence the WT and mutant-associated regions to verify the authenticity of the mutant loci. The sequences were also aligned using MAGA11^2^ to detect the variants. Moreover, CsRST1 proteins and homologous sequences of different species were downloaded from the NCBI database to verify the conservation of protein sequences. The downloaded RST1 proteins were *Cucumis sativus* (CsRST1, accession No. XP_011652289.1), *C. melo* (CmRST1, accession No. XP_008443559.1), and *Benincasa hispida* (BhRST1, accession No. XP_038904941.1). The others were from *Shorea leprosula* (SlSLEP1 accession No. GKV07771.1), *Jatropha curcas* (JcRST1, accession No. XP_020534238.1), rubber (*Hevea brasiliensis*; HbRST1, accession No. XP_021686448.1), *Quercus suber* (QsRST1, accession No. XP_023891260.1), and *Q. lobata* (QlRST1, accession No. XP_030953860.1).

The cotyledons of three SC311 (WT) and three *v-3* plants were analyzed to validate the transcriptome data. Briefly, total RNA was extracted using the TRIzol Reagent (Invitrogen, Carlsbad, CA, United States) and reverse transcribed to cDNA using a PrimeScript™ RT Kit (Takara Bio, Inc., Shiga, Japan) following the manufacturer’s instructions. Actin was the internal reference gene, and the 2 ^−△△CT^ method was used to calculate the relative gene expression.

## Results

### Phenotypic characteristics of the *v-3* mutant

**Figure 1:**
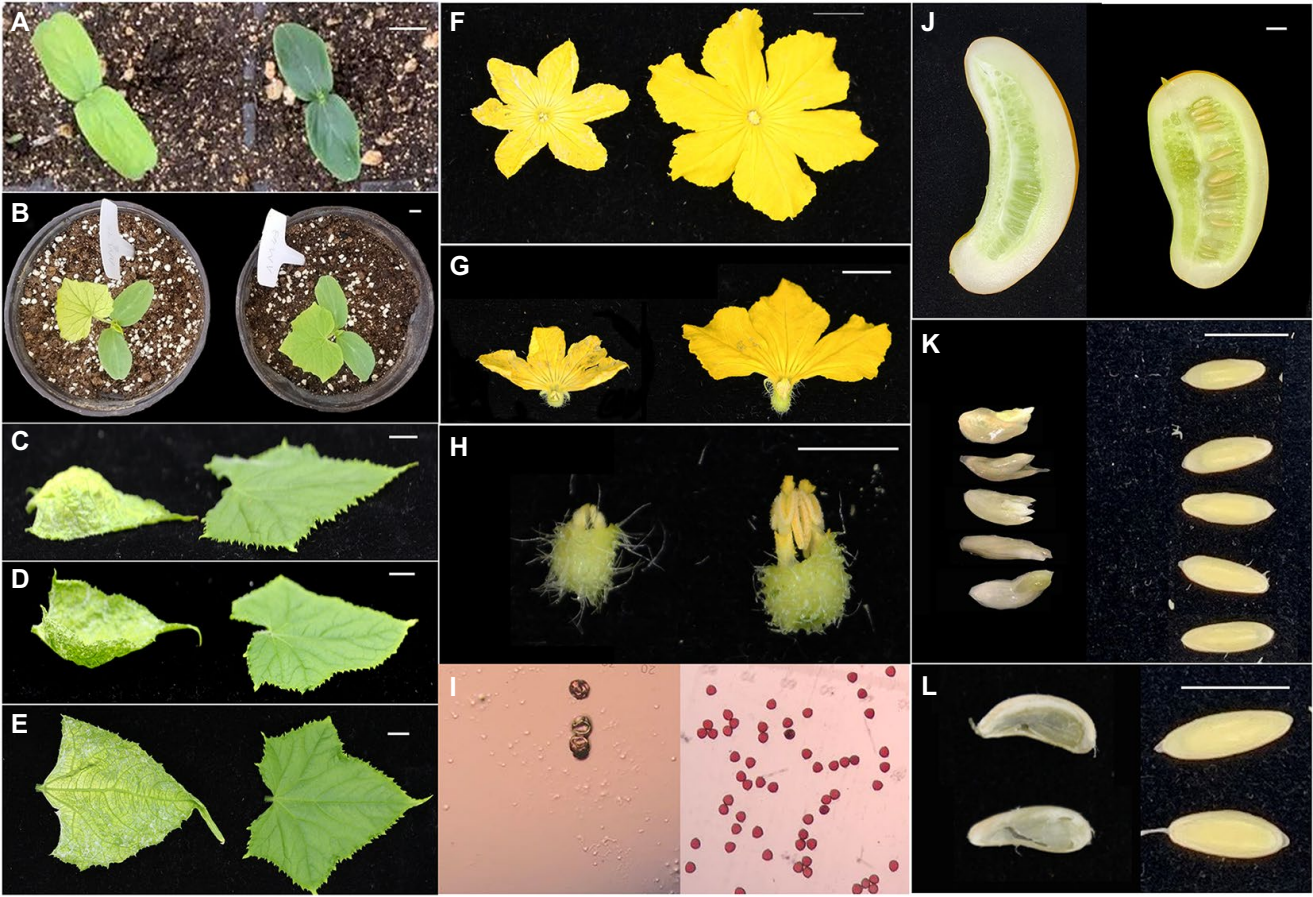
Phenotypic analysis of the *v-3* mutant. **(A)** Cotyledon color of *v-3* and the WT. **(B)** True leaf color of *v-3* and the WT. **(C)** Upward convex leaf of *v-3* and normal leaf of the WT. **(D)** Downward concave leaf of *v-3* and normal leaf of the WT. **(E)** White hyaline cracks of *v-3* and normal leaf of the WT. **(F)** Male flower of *v-3* and the WT. **(G)** Male flower section of *v-3* and the WT. **(H)** Stamen of *v-3* and the WT. **(I)** Pollen of *v-3* and the WT. **(J)** Section of fruit after selfing of *v-3* and the WT. **(K)** Seeds after hybridization of *v-3* and the WT. **(L)** Testa after hybridization of *v-3* and the WT. Scale bar = 1 cm. WT, wild type.

The yellow-leaf *v-3* mutant is a spontaneous mutant isolated from the selfed cucumber line SC311 that was originally from India. The cotyledons (Figure 1A) and true leaves (Figure 1B) of *v-3* are initially yellow and gradually turn green. The duration of turning green varies with light and temperature conditions. At low temperatures or light intensity, the chlorophyll content of the yellow-turned-green leaves was significantly lower than that of the wild plants. The first and second true leaves of *v-3* are flat; the third and fourth true leaves are variable, but from the fifth (±2) true leaf onwards, the base of the true leaf grows abnormally (Figure 1E), causing the leaf to bulge upward (Figure 1C) or depress downward (Figure 1D). The leaves also developed white hyaline cracks (Figure 1E). However, the *v-3* mutant male flowers were abnormal, since some male flowers only formed buds and directly withered without flowering. The flowers of flowering males were small (Figure 1F) with shrunken anthers in their stamens (Figures 1G,H), and failed to form fertile pollen (Figure 1I). Nonetheless, the female flowers of the mutant matured and flowered normally, and the fruits expanded after parthenocarpy, although they failed to form seeds (Figure 1J). The seeds formed from crosses with WT and normal pollen were abnormally shaped (Figure 1K), had incomplete seed coats (Figure 1L), and had abnormal embryo-morphology, although they developed into complete plants.

**Figure 2:**
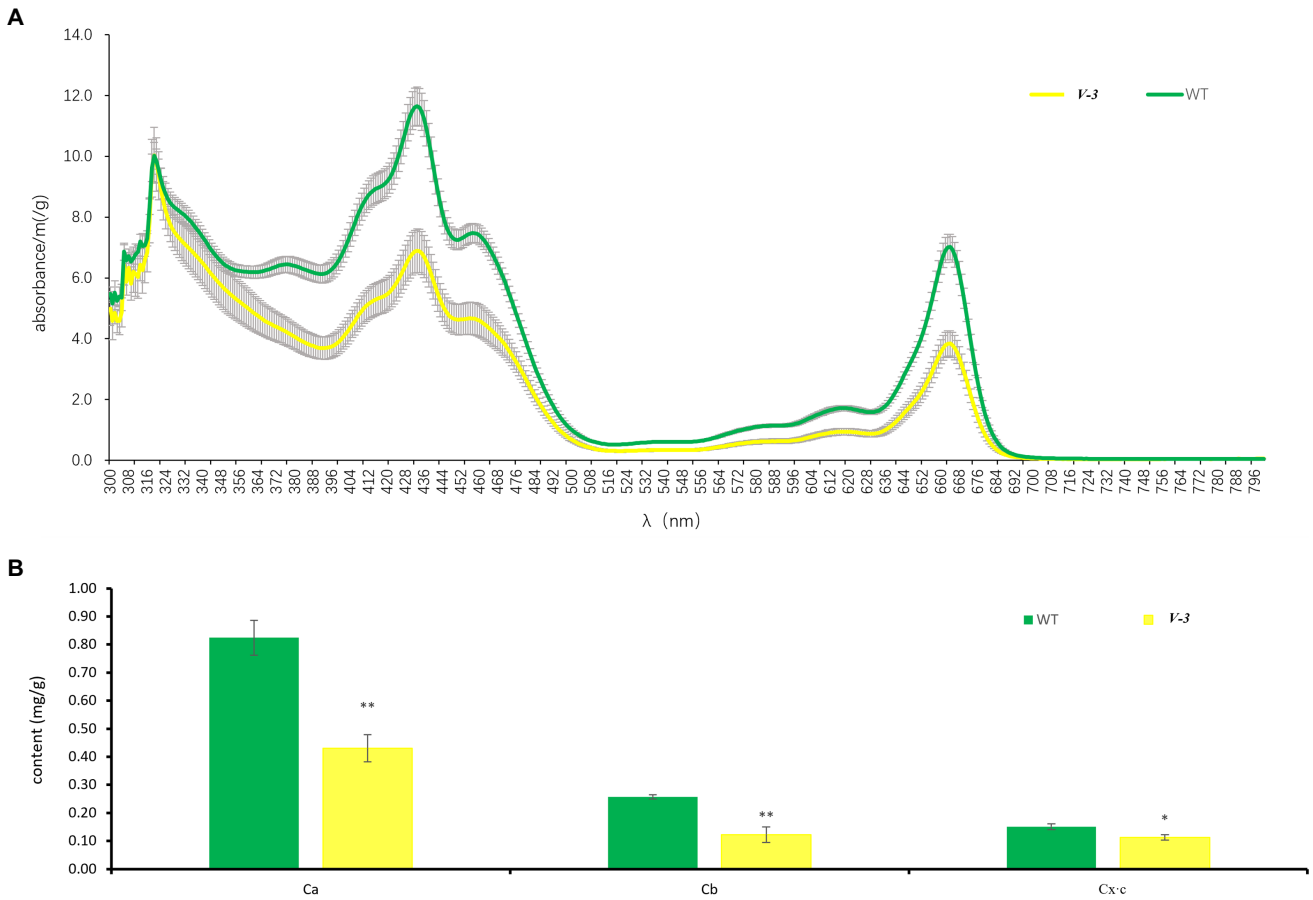
Leaf pigments of *v-3* and the WT. **(A)** The spectral scans of leaf pigments of *v-3* and the WT. **(B)** Chlorophyll a, chlorophyll b, and carotenoid content of *v-3* and the WT. Data are the mean ± SD. Error bars represent the SD of five independent replications. SD, standard deviation; WT, wild type. ^*^p < 0.05. ^**^p < 0.01.

The SC311 (WT) and *v-3* mutants differed greatly in color, but the spectral scans of leaf pigments from SC311 (WT) and *v-3* mutants were similar, and the highest absorption wavelengths were the same. SC311 (WT) had a higher absorption peak. The leaf pigment fraction of mutant *v-3* did not significantly change but decreased (Figure 2A). The pigment content was significantly lower in the mutant than in the WT, with 52.2, 47.7, and 74.5% chlorophyll a, chlorophyll b, and carotenoid contents of SC311, respectively (Figure 2B).

### *v-3* is a light-sensitive and thermo-sensitive mutant

**Figure 3:**
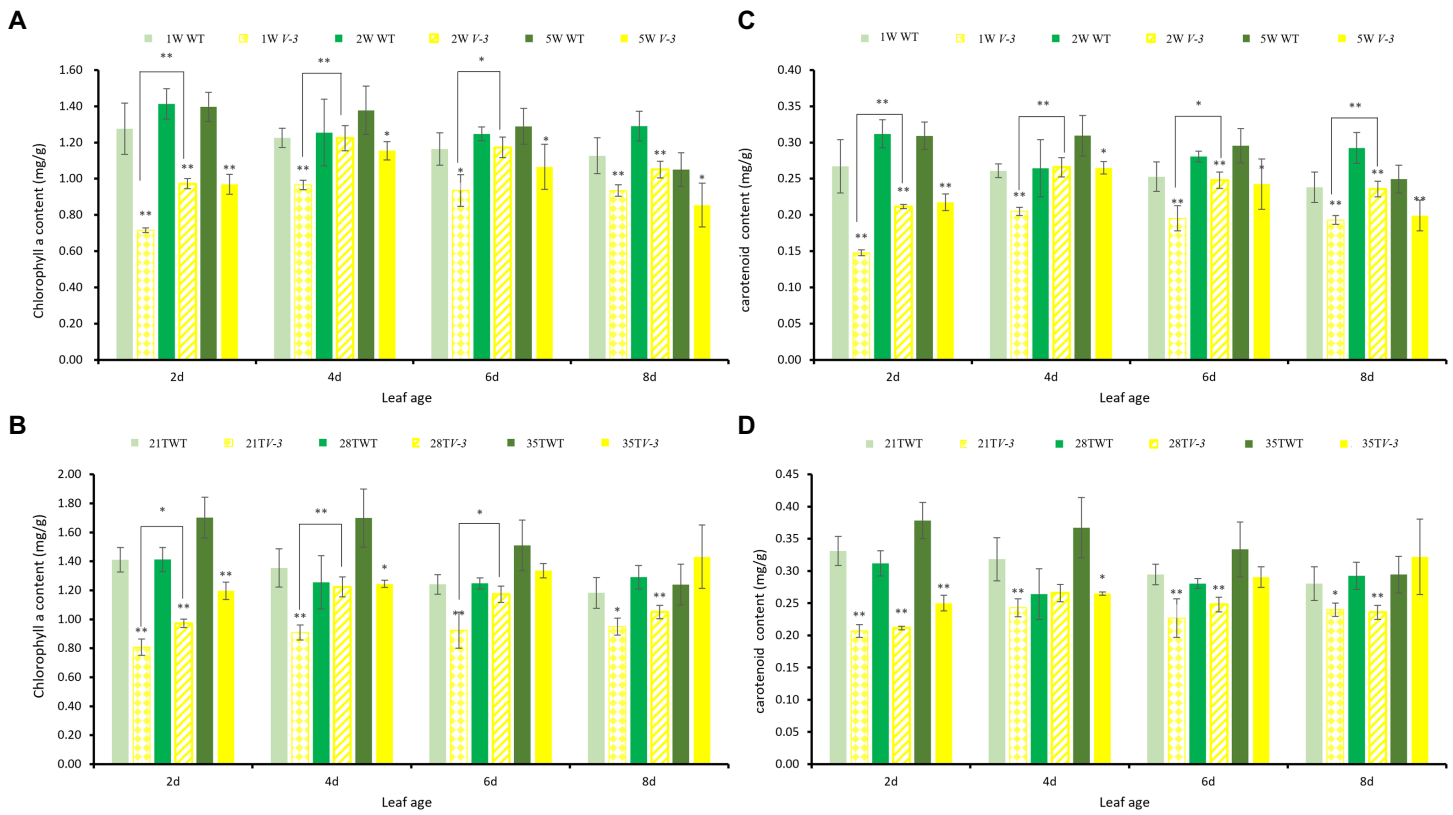
The leaf pigment contents of *v-3* and the WT under different light intensity and temperature treatments. **(A)** Chlorophyll a content of *v-3* and the WT under different light intensity treatments. **(B)** Chlorophyll a content of *v-3* and the WT under different temperature treatments. **(C)** Carotenoid content of *v-3* and the WT under different light intensity treatments. **(D)** Carotenoid content of *v-3* and WT under different temperature treatments. Values are the mean ± SD. SD, standard deviation; WT, wild type. ^*^p < 0.05. ^**^p < 0.01.

The pigment contents of cotyledons at different leaf ages were determined under the 1W28T, 2W28T, and 5W28T treatments to determine the response of the mutants to light intensity. Compared with 1W28T, the chlorophyll a content of the WT plants under the 2W28T treatment increased by 10.8, 2.4, and 7.2% at 2, 4, and 6 days, respectively. However, none of them were significant. In contrast, the chlorophyll a content in the *v-3* plants increased significantly by 35.9, 26.7, and 25.4%, but the high-intensity light (50,000 Lx) was not conducive to the accumulation of pigments (Figure 3A). The contents of chlorophyll b (Supplementary Figure 1), carotenoids (Figure 3C), and chlorophyll a changed consistently, and the true leaves at different leaf ages consistently responded to different light intensities (data not shown). Therefore, mutant *v-3* is a light-sensitive mutant.

The cotyledon pigmentations at different ages were measured under the 2W21T, 2W28T, and 2W35T treatments to determine the response temperature. Compared with 2W21T, the chlorophyll a content of the WT plants under 2W28T was the same at 2, 4, and 6 days, but the contents in the *v-3* mutant significantly increased by 20.4, 34.7, and 26.8%, respectively (Figure 3B). Chlorophyll b and chlorophyll a changed consistently (Supplementary Figure 2), but the carotenoid content did not differ (Figure 3D). The true leaves at different ages responded consistently to different temperatures (data not shown). Thus, mutant *v-3* is thermo-sensitive.

### *v-3* has low photosynthetic efficiency

**Figure 4:**
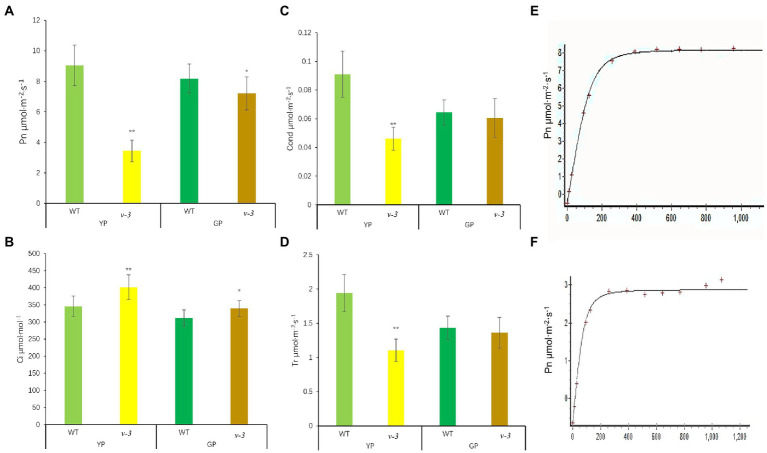
Photosynthetic parameters of *v-3* and the WT in yellowing period (YP) and turning green period (GP). **(A)** Net photosynthetic rate (Pn); **(B)** Intercellular CO_2_ concentration (Ci); **(C)** Stomatal conductance (Gs); **(D)** Transpiration rate (Tr); **(E)** light response curve of WT; **(F)** light response curve of *v-3*; Values are the mean ± SD. SD, standard deviation; WT, wild type. ^*^p < 0.05. ^**^p < 0.01.

The net photosynthetic rate (Pn) of *v-3* was only 38.1% of that of SC311 (WT; Figure 4A). However, the intercellular CO_2_ concentration in *v-3* was significantly higher by 16.1% than that of SC311 (WT; Figure 4B), indicating that the decreased Pn was due to non-stomatal causes. The photosynthetic capacity gradually recovered after *v-3* turned green, reaching 88.2% as in SC311 (Figure 4A). The transpiration rate and stomatal conductance also showed similar changes (Figures 4C,D). The light compensation point of the *v-3* mutant increased significantly, and the apparent photometric quantum efficiency (AQE) and light saturation point were significantly reduced by 33.9 and 38.7% (Figures 4E,F).

### Differences in chlorophyll fluorescence of *v-3* predicts variation in the PSII structure

The chlorophyll fluorescence of the mutant and SC311 (WT) leaves at the same leaf age [yellowing (YP) and turning green periods (GP)] was measured to study the differences in the photosynthetic capacity of the mutant and WT. The dark adaptation was utilized to completely open the photosystem II (PSII) reaction center to attain the maximum photochemical quenching value. Fo is the dark fluorescence yield measured after plant adaptation to the dark, a purely physical process that is independent of the photosynthetic light reaction (Krause and Weis, 1984). After the mutants turned green, the WT Fo remained unchanged, while the mutant Fo decreased. The Fo of the yellow and greening mutants were significantly higher than that of the WT (Table 1), indicating that there were structural variations in the PSII in the mutants (Demmig et al., 1987).

**Table 1:** Chlorophyll fluorescence parameters of WT and *v-3*.

	Fo^1^	ETR^2^	NPQ^3^	qL^4^	qN^5^	qP^6^	Y(II)^7^	Y(NPQ)^8^	Fv/Fm^9^	Fv/Fo^10^
WT(YP)	0.133 ± 0.007	20.778 ± 1.156	0.232 ± 0.020	0.394 ± 0.021	0.608 ± 0.024	0.619 ± 0.020	0.371 ± 0.015	0.309 ± 0.019	0.752 ± 0.021	3.035 ± 0.200
*v-3*(YP)	0.177 ± 0.013^**^	11.394 ± 2.131^**^	0.337 ± 0.026^**^	0.275 ± 0.051^**^	0.756 ± 0.049^**^	0.402 ± 0.096^**^	0.174 ± 0.049^**^	0.475 ± 0.043^**^	0.649 ± 0.032^**^	1.845 ± 0.270^**^
WT(GP)	0.131 ± 0.003	17.853 ± 2.315	0.294 ± 0.023	0.322 ± 0.076	0.665 ± 0.015	0.539 ± 0.074	0.320 ± 0.041	0.368 ± 0.025	0.761 ± 0.026	3.179 ± 0.462
*v-3*(GP)	0.165 ± 0.024^**^	15.556 ± 2.277	0.242 ± 0.089^*^	0.271 ± 0.086	0.646 ± 0.129	0.442 ± 0.122	0.235 ± 0.041	0.375 ± 0.110	0.714 ± 0.050	2.776 ± 0.499

Values are the mean ± SD. SD, standard deviation.

* *p* < 0.05;

** *p* < 0.01.

1 Initial fluorescence.

2 Electron transfer rate.

3 Non-photochemical quenching coefficient.

4 Photochemical quenching coefficient.

5 Non-photochemical quenching coefficient.

6 Photochemical quenching coefficient.

7 Actual quantum yield of PSII.

8 Quantum yield of regulated energy dissipation.

9 Maximum quantum yield of PSII.

10 Potential PSII activity.

The F_v_/F_m_ is the maximum quantum yield of PSII, which reflects the potential maximum photosynthetic capacity of the plant and represents the PSII primary light energy conversion efficiency. The F_v_/F_m_ of mutant was significantly lower than the WT at the YP, although the Fv/Fm increased at the GP of the mutant. Nonetheless, the potential photosynthetic capacity of the mutant was also lower than that of the WT (Table 1). The parameter Fv/Fo represents the potential PSII activity (Lu et al.,1993). The difference between mutant and WT Fv/Fo was similar to the F_v_/F_m_. Therefore, the F_v_/F_m_ and Fv/Fo differences indicate the structural abnormalities in PSII.

Y(II) is the actual quantum yield of PSII, which reflects the actual photosynthetic efficiency of the plant. The Fv/Fm of the mutant was significantly lower (only 47.0% of the WT) at the YP. Moreover, the Fv/Fm of the mutant remained significantly lower than that of the WT (73.4%) at the GP. The photosynthetic electron transfer rate (ETR), another indicator of photosynthetic efficiency, was measured. The ETR of the mutant was 54.8% of the WT in the YP, although it improved to 87.1% of the WT in the GP. The results of these two chlorophyll fluorescence parameters indicated that the photosynthetic efficiency was consistent with that determined by the LI-6400XT portable photosynthesis system. The photosynthetic efficiency of the mutant was significantly lower than the WT at YP but was close to the WT at GP (Table 1).

The fluorescence quenching caused by photosynthesis, which is designated photochemical quenching (qP/qL), reflects the level of photosynthetic activity in the plants. The qP establishes the PSII antenna pigment system as the “swamp model,” and the qL establishes the PSII antenna pigment system as the “lake model.” In this study, the photochemical quenching of both models was significantly lower in the yellowing mutant than in the WT (Table 1). Moreover, the fluorescence quenching caused by thermal dissipation is called non-photochemical quenching (qN or NPQ). Non-photochemical quenching was significantly higher in the mutant than the WT at the YP stage. The quantum yield of regulatory energy dissipation Y(NPQ) in the mutant was significantly greater than that of the WT at YP. Thus, the mutant has a severe excess of light energy, and the plant can protect itself by dissipating the excess light energy through its regulatory mechanism (Table 1).

### Abnormal chloroplast structure in *v-3* leaves

**Figure 5:**
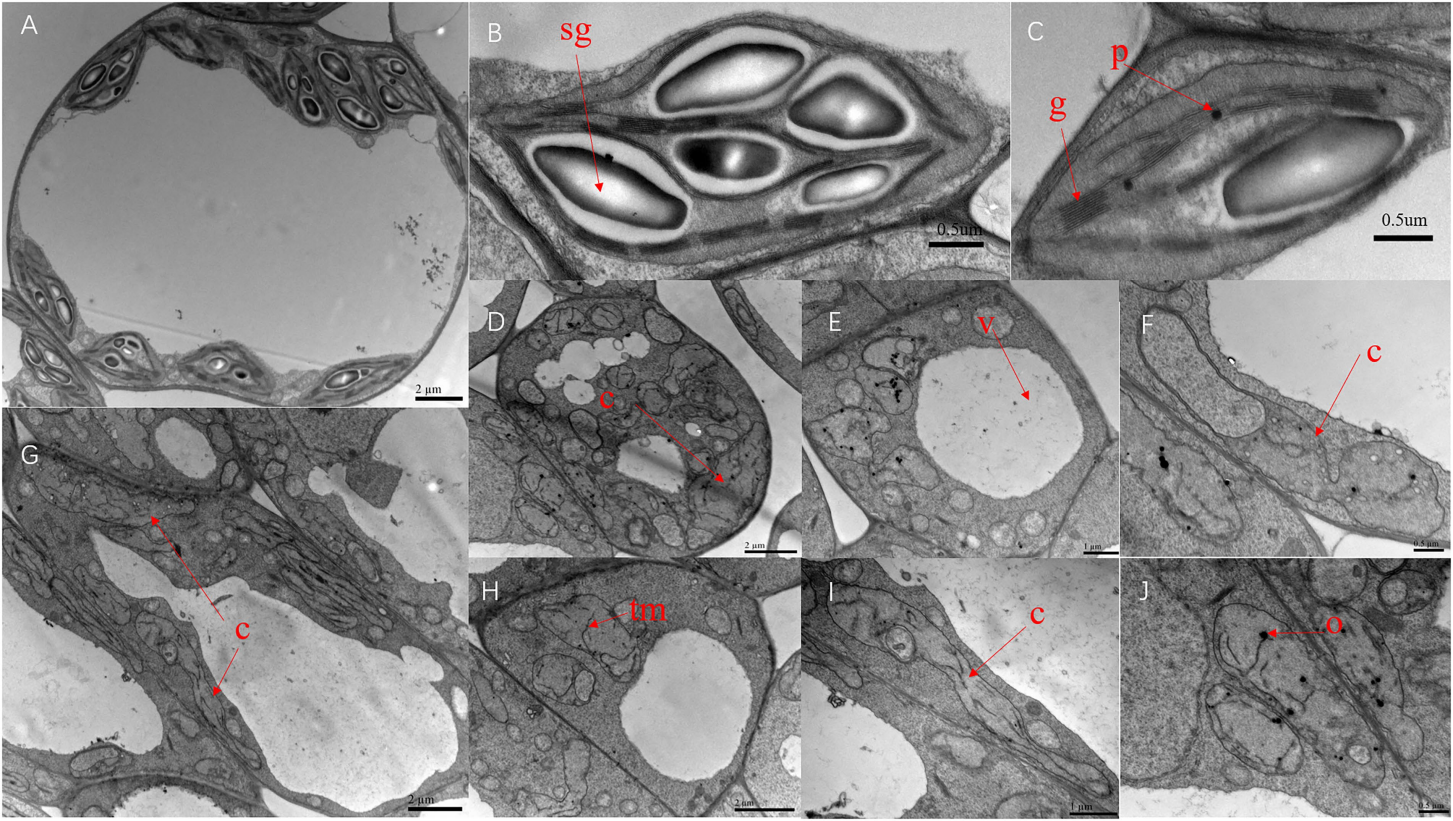
Transmission electron microscopy of the chloroplast ultrastructure of SC311 **(A–C)** and *v-3* **(D–J)**. Sg, starch grain; G, grana; O, osmiophilic granule; TM, thylakoid membrane; V, vesicle; and C, chloroplast.

The ultrastructure of SC311 (WT) and *v-3* chloroplasts were observed during the first true leaf period using TEM to investigate the effect of the *v-3* mutation on chloroplast structure. The WT chloroplasts were full, uniformly sized, and shaped like an olive ball, with a short and thick morphology (Figure 5A). However, the WT chloroplasts had large vesicles (Figure 5A), contained abundant starch granules (Figure 5B) and neatly stacked grana, and had few osmiophilic granules (Figure 5C). The mutant chloroplasts had distorted thylakoid membranes (Figures 5E,G,H), no grana, scarce starch granules, and abundant osmiophilic granules (Figures 5D,E,H,J). Moreover, the mutants had few chloroplasts (Figures 5E,G,H) that had an aberrant morphology (Figures 5D–J). Chloroplasts in the same cells of the mutant leaves varied greatly in size (Figures 5D,G). There were only a few chloroplasts, and they were unusually large (Figures 5D,F,G,I,J), indicating abnormal chloroplast division.

### Genetic characteristics of *v-3*

The heterozygous green SC311 was self-crossed to obtain the F_2_-A population. Since the mutant was male sterile, the green plant “9930” was the father, and mutant *v-3* was the mother for generating the F_1_ that was crossed to obtain the F_2_-B population. The 77 plants of the F_2_-A population had 59 green and 18 yellow plants, corresponding to the 3:1 ratio of Mendel’s law of segregation (χ^2^ = 0.11, *p* = 0.74). Moreover, the 93 plants of the F_2_-B population had 73 green and 20 yellow plants, also corresponding to the 3:1 ratio of Mendel’s law of segregation (*χ^2^* = 0.61, *p* = 0.74). These results suggest that a single recessive nuclear gene controls the *v-3* mutant phenotype.

### Fine mapping of the *v-3* locus

**Figure 6:**
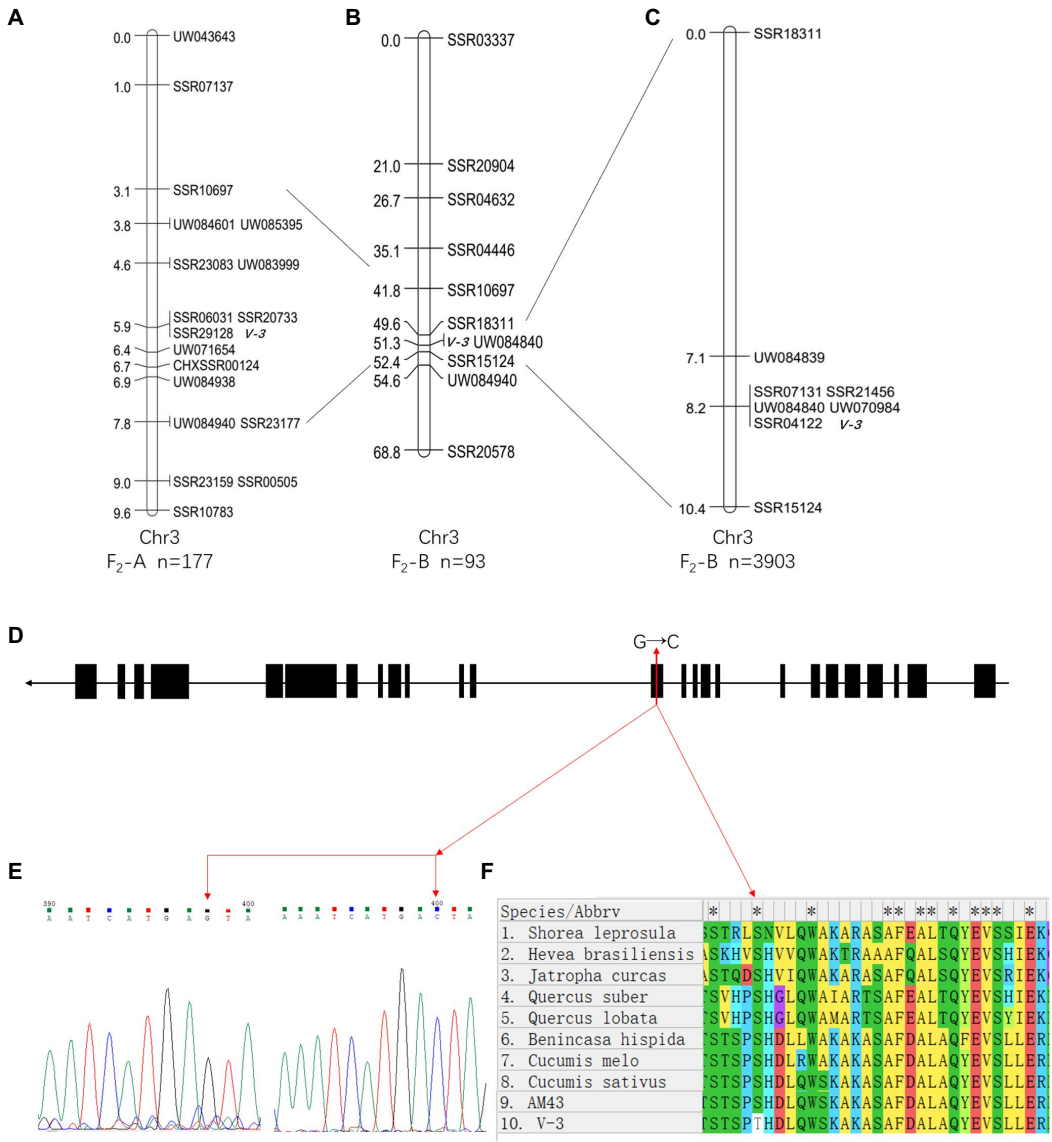
Positional cloning and sequence alignment of the *v-3* gene. **(A)** Numbers on the chromosome left are genetic distance (cM). **(B)** Mapping based on 93 plants from the F_2_-B population placed the *v-3* locus on chromosome 3 at a 2.8 cM region. **(C)** Mapping based on 3,903 plants from the F_2_-B population placed at the *v-3* locus between UW084839 and SSR15124. **(D)** Structure of the *v-3* candidate gene, which has 25 exons. The SNP (G → C) in exon 13 changes serine to threonine. **(E)** Sequencing peak map of the SNP sites in *v-3* and the WT. **(F)** Protein sequence alignment of the RST1 gene in “SC311,” “*v-3*,” “*Cucumis sativus*,” “*C. melo*,” “*Benincasa hispida*,” “*Shorea leprosula*,” “*Jatropha curcas*,” “*Hevea brasiliensis*,” “*Quercus suber*,” “*Q. lobata*.” ^*^a conservative sequence. Black boxes and black lines indicate exons and introns, respectively. SNP, single nucleotide polymorphism.

SC311 and *v-3* had 18 polymorphic SSR markers (8.2%) from 220 evenly distributed primers on the seven chromosomes. A BSA strategy was used to genetically map *v-3*. The green and yellow leaf pools were used as templates, and the 18 polymorphic markers screened in the previous step were used to identify the markers linked to the mutant. Therefore, three markers were associated with *v-3* (UW043643, SSR20733, and SSR10783). Next, 107 SSR markers were developed in this region, and 15 polymorphic markers were detected to pinpoint the location of *v-3* in more detail. The population was expanded to 177, including the previous 60 plants. We constructed the genetic map that showed that *v-3* is located between UW083999 (chr3, 32156194) and UW071654 (chr3, 35644875), with a physical distance of 3.49 Mb, and it co-segregated with SSR20733, SSR29128, and SSR06031 (Figure 6A).

In the F_2_-B population, the 220 SSR markers were evenly distributed over seven chromosomes, with 64 polymorphic SSR makers (29.1%) between 9930 and *v-3*. A total of 93 individuals were randomly selected from the F_2_-B population, and individual genotypes were identified using the polymorphic markers from the previous step. The genetic maps were constructed using JoinMap 4.0 (Figure 6B). Mutant *v-3* was mapped between SSR18311 and SSR15124 on chromosome 3 and was linked to UW084840. The two flanking markers lacked recombination events, so the population was expanded to 647 plants, including the starting 93 plants. Next, 49 SSR markers were developed to narrow the target region, and four were polymorphic markers. A local genetic map was constructed with the polymorphic markers, two flanking markers, and the co-segregating marker (UW084840). The two flanking markers, UW084839, and SSR15124 localized *v-3* to 3.3 cM (~2.1 Mb). Moreover, there were five co-segregating markers (SSR21456, SSR04122, UW070984, UW084840, and SSR07131). Since there was no recombination event, the population was expanded to 3,903 plants, including the previous 647 plants. Again, there was no recombination event (Figure 6C), presumably because this position was the cold spot for recombination, and the interval could have been inverted. Combined with the results of the F_2_-A population, *v-3* was finally mapped to UW084839 (chr3, 33536014) and SSR15124 (chr3, 35661331).

### BSA whole-genome resequencing

**Figure 7:**
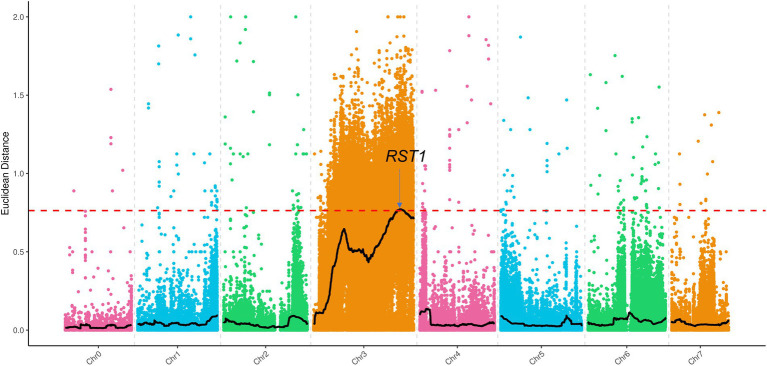
BSA-Seq approach for mapping the *v-3* locus. The black lines show the results of ED, and the red lines show the threshold of ED. ED, Euclidean distance.

A G-pool and Y-pool of each 15 extremely green and yellow plants were selected from the F_2_-A population, respectively, using the BSA-Seq strategy. A total of 30.81 and 24.87G valid bases were obtained from the G-pool and Y-pools on the Illumina NovaSeq 6000 sequencing platform, respectively. With “Gy14” as the reference genome, the average depth of coverage was 94.9 and 74.76 from the G-pool and Y-pools, respectively. Next, SNPs of the G-pool and Y-pools were obtained by GATK, and the regions associated with *v-3* were identified using the ED algorithm (Figure 7). The localization regions are shown in Supplementary Table 2. The region obtained by BSA-Seq was consistent with the linkage region. Finally, *v-3* was mapped to the 1.34 Mb region of chromosome 3 (Supplementary Table 3).

### Candidate gene identification

A total of 106 genes were annotated in the 1.34 Mb region using “Gy14” genome sequence data. The SNPs of the 106 genes were identified based on the re-sequencing data and analyzed under the following conditions: heterozygous in the G-pool; homozygous in the Y-pool, and mutations in the Y-pool were inconsistent with the reference genome but were nonsynonymous. Ten genes with 22 SNPs were found to be eligible, and their specific results are shown in Supplementary Table 4. None of these 10 genes were DEGs, but *CsaV3_3G042730* was identified when “9930” was the reference genome. *CsaV3_3G042730* was a DEG with SNP that met screening conditions, and all the DEGs in this interval are shown in Supplementary Table 5. In addition, no SNP that met the screening conditions existed upstream of *CsaV3_3G042730*, thus, confirming that the differential expression was not due to variation in the promoter. The “Gy14” and “9930” reference genomes showed consistent DNA sequences between the two genomes, except for the length of the 13th exon. The online annotation tool FGENESH^3^ predicted results consistent with “9930” (Supplementary Figure 3). In addition, clone sequencing (Supplementary Figure 4) also confirmed that the missing portions of exon 13 and the SNP site are actually present (Figure 6E). Gene *CsaV3_3G042730* is 5568 bp long with 25 exons, but a single nucleotide mutation (G → C, serine to threonine) occurred in exon 13 (Figure 6D). Furthermore, we compared the missing portions with the protein sequences of other plants to determine the amino acid conservation. The results showed that serine was conserved (Figure 6F). Thus, it was identified as a candidate gene. *CsaV3_3G042730* encodes the RST1 protein, and the *rst1* mutant in *Arabidopsis* has abnormal leaf shape, late inflorescence appearance, and nonviable seeds (Chen et al., 2005). RNA exosomes and RST1 form high-order complexes that catalyze the maturation and degradation of chloroplast RNA (Lange and Gagliardi, 2022; Lee and Suh, 2022).

### Comparative transcriptome analysis of SC311 and *v-3*

RNA-Seq was performed on the flattened cotyledons of SC311 and *v-3* to reveal the molecular mechanisms of the virescent leaf. Thus, 10 genes were randomly identified, and their relative level of expression in flattened cotyledons of SC311 and *v-3* was determined to validate the transcriptome results. The results from real-time quantitative reverse transcription (RT-qPCR) and RNA-Seq were consistent, showing similar expression patterns and an excellent Pearson correlation (*r* > 0.96), indicating high reliability of the RNA-Seq results (Supplementary Figure 5).

SC311 and *v-3* generated 1,104 DEGs, including 643 upregulated and 461 downregulated genes (Supplementary Figure 6). GO annotation and functional enrichment indicated that the DEGs were primarily enriched in terms of DNA-binding transcription factor activity [molecular_function (MF)], cysteine-type endopeptidase inhibitor activity (MF), DNA binding (MF), the transcription factor complex [cellular_component, (CC)], and response to biotic stimulus [biological_process (BP); Supplementary Figure 7]. Thus, the functions of DEGs are related to transcription.

The KEGG pathway enrichment showed that the downregulated genes were primarily enriched in ribosome biogenesis in eukaryotes, which had the highest number and value of enriched genes (Supplementary Figure 8). The upregulated genes were primarily enriched in ribosome (having the highest number of enriched genes) and porphyrin metabolism (having the highest value of enriched genes; Supplementary Figure 9). The two largest components in the KEGG pathway classification were transcription and carbohydrates. The carbohydrates could be due to the enormous difference in photosynthesis that resulted in different carbohydrate contents. Both GO and KEGG enrichment analyses supported the hypothesis that the candidate gene is regulatory RNA.

**Figure 8:**
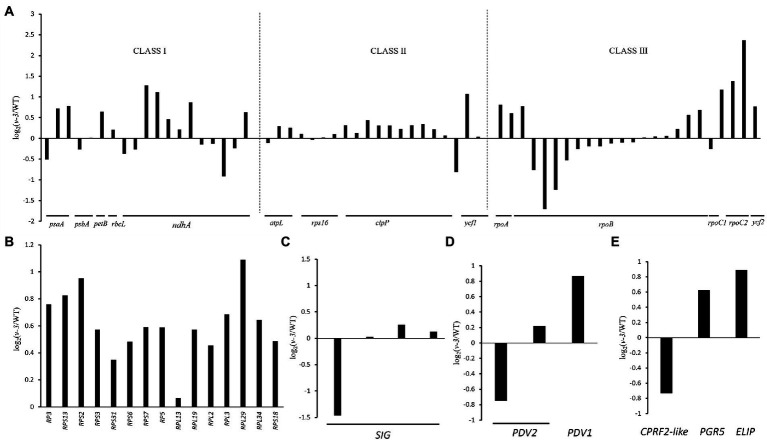
Transcriptional changes of chloroplast-related genes between the WT and *v-3* based on RNA-Seq data. **(A)** Levels of gene expression controlled by different polymerases, Class I: PEP, Class II: PEP and NEP, and Class III: NEP. **(B)** The levels of expression of rRNAs in the WT and v-3. **(C)** The levels of expression of *SIG* in the WT and v-3. **(D)** The levels of expression of *PDV1* and *PDV2* in the WT and *v-3*. **(E)** The levels of expression of *CPRF2-like*, *PGR5*, and *ELIP* in the WT and *v-3*. NEP, nuclear-encoded RNA polymerase; PEP, plastid RNA polymerase; CPRF2, common plant regulatory factor 2; PGR5, Proton gradient regulation; ELIP, Early light-induced protein; WT, wild type.

Since the mutant is thermo-and light-sensitive, three genes co-expressed with the candidate genes were identified through the transcriptome (Figure 8E), and the mutant could be responding to light and temperature through these three genes. *CsaV3_3G012890* that encoded Early light-induced protein (ELIP) is one of the earliest genes induced during photomorphogenesis (Harari-Steinberg et al., 2001), which responds to temperature (Peng et al., 2008) and light intensity (Hutin et al., 2003). *CsaV3_1G039550* encodes the light-inducible protein CPRF2 (common plant regulatory factor 2)-like, a basic region/leucine zipper motif (bZIP) transcription factor (Jakoby et al., 2002) that responds to temperature (Feng et al., 2018) and light intensity (Weisshaar et al., 1991). *CsaV3_3G011780* encodes Proton gradient regulation 5 (PGR5) in response to light intensity (Suorsa et al., 2012; Kameoka et al., 2021). The mutant might receive light and temperature signals through these three genes.

**Figure 9:**
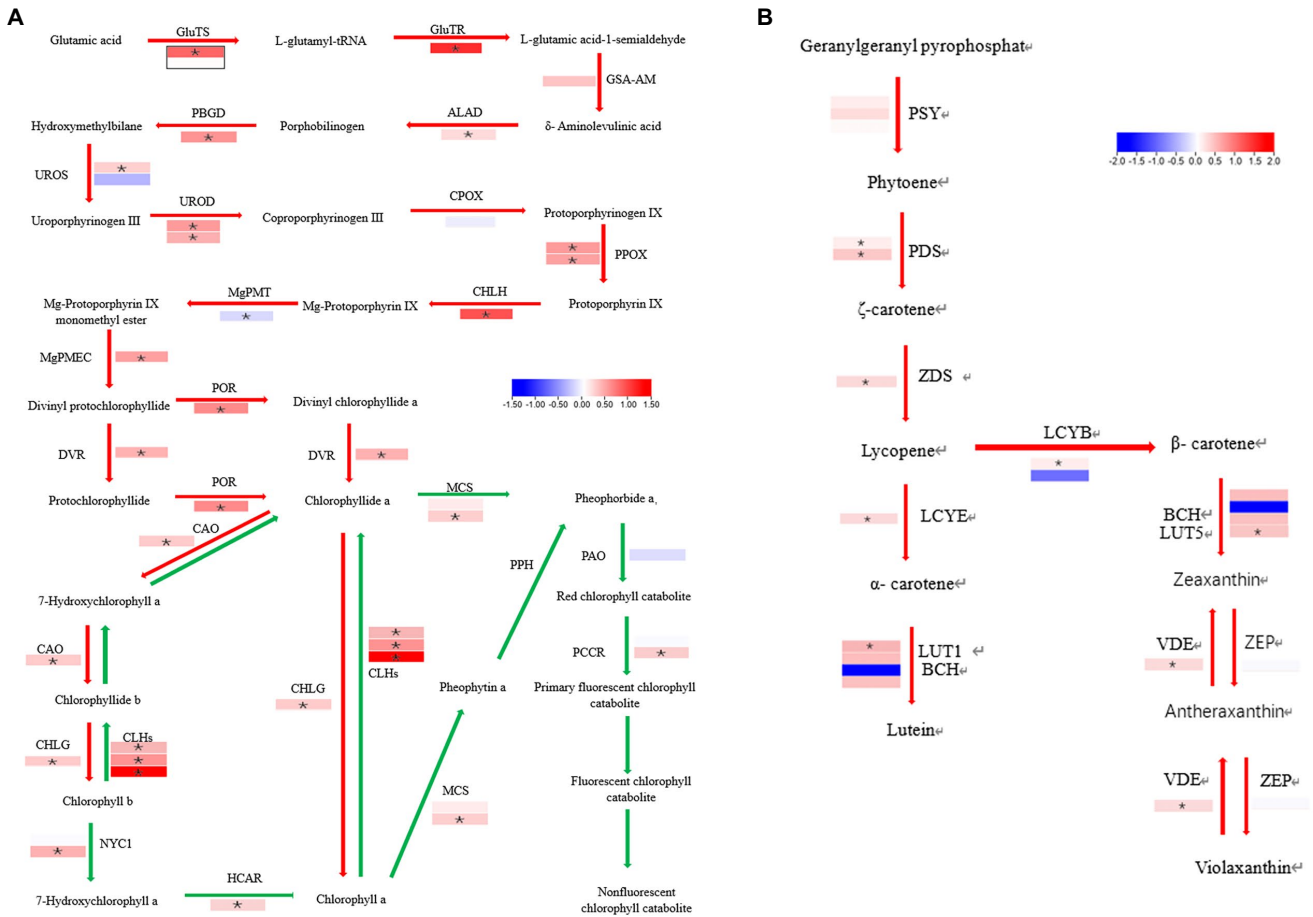
Difference in the enzyme expression between the WT and *v-3* in the chlorophyll and carotenoid biosynthetic pathways. **(A)** The red arrow represents the chlorophyll synthesis pathway, and the green arrow represents the chlorophyll decomposition pathway. **(B)** Carotenoid biosynthesis pathway. In mutants, the upregulated gene is in red boxes, and the downregulated gene is in blue boxes. The white boxes represent genes with unchanged expression. ^*^DEGs with | FoldChange| > 1.5 and *p* < 0.05. DEGs, differentially expressed genes; WT, wild type.

The chlorophyll biosynthetic catabolic (Beale, 2005) and the carotenoid biosynthetic (Cunningham, 2002) pathways were investigated to identify the causes of the virescent leaf. No enzyme was significantly down-regulated in the chlorophyll biosynthetic pathway, the genes are listed in Supplementary Table 6. The gene for chlorophyllase, CLHs (*CsaV3_2G013440*, *CsaV3_5G025230*, and *CsaV3_2G013640*) was significantly up-regulated in the chlorophyll catabolic pathway in the mutant (Figure 9A). The expression of lycopene β-cyclase (*CsaV3_4G000740*) was reduced but insignificant in the carotenoid biosynthetic pathway (Figure 9B).

Since chloroplast synthesis is jointly regulated by plastid RNA polymerase (PEP) and nuclear-encoded RNA polymerase (NEP), three groups were selected to investigate the effect of mutants on both types of regulation: The first group, which was controlled by PEP, contained *PsaA*, *PsbA*, *PetB*, *rbcL*, and *ndhA*; The second group, which was jointly controlled by NEP and PEP, contained *rps16*, *clpP*, *atpl*, and *ycf1*; The third group, controlled by NEP, contained *RpoA*, *RpoB*, *RpoC1*, *RpoC2*, and *ycf2* (Chateigner-Boutin et al., 2008; Myouga et al., 2008). The genes in each of the three groups were both up- and downregulated, the genes are listed in Supplementary Table 7. The results showed that *v-3* does not regulate specific transcriptional processes (Figure 8A). Similarly, all the ribosome-related genes were up-regulated in the mutants, the genes are listed in Supplementary Table 8. Thus, virescent leaf was not due to the downregulated expression of the chloroplast ribosome (Figure 8B). However, the *SIG* that encodes the PEP σ factor (Figure 8C) and the *plastid division1 (PDV1)* that encodes the plastid division protein were significantly reduced (Figure 8D), the genes are listed in Supplementary Table 9. The nuclear-encoded σ factor is a key to controlling the binding of RNA polymerase to the promoter in chloroplasts (Lysenko, 2007). However, the expression of genes regulated by PEP was not reduced. The plastid division proteins, PDV1 and plastid division2 (PDV2), form a complex and are the primary mechanical determinants of chloroplast numbers (Miyagishima et al., 2006; Okazaki et al., 2009). We hypothesized that the mutant may regulate chloroplast development by modulating *PDV2*, leading to virescent leaf, considering the abnormal chloroplast division observed in the mutant.

## Discussion

Photosynthesis, an important chemical reaction on earth, uses sunlight to produce the oxygen needed for most life activities while providing energy to plants. Chloroplasts are the material basis for photosynthesis, and variations in the chloroplast structure can affect leaf color and photosynthesis. Normally, chloroplasts are found in plants, algae, and protozoa. However, leaf color mutations are associated with chloroplast structural variations in several plants, including rice (Chen et al., 2018), Arabidopsis (Song et al., 2004), tobacco (Bae et al., 2001), pepper (*Capsicum annuum*; Shi et al., 2022), maize (Schultes et al., 1996), tomato (*Solanum lycopersicon*; Chang et al., 2021), and wheat (*Triticum aestivum*; Li et al., 2013). In this study, the chlorophyll a, chlorophyll b, and carotenoid contents of the mutants were significantly reduced, but the pigment composition was consistent with that of the WT.

In addition, the Pn of mutant was significantly lower than that of the WT. The stomatal conductance of the mutant was significantly lower than the WT but remained substantially high. In contrast, the intercellular CO_2_ concentration of the mutant was significantly higher than that of the WT. The abnormal chloroplast structure seriously reduced the ability of assimilating CO_2_ despite its reduced stomatal conductance, resulting in a higher intercellular CO_2_ concentration. Therefore, non-stomatal factors reduced the photosynthetic rate of the mutant due to the structural abnormalities of the chloroplast.

Chlorophyll fluorescence, gas exchange, and photosynthetic oxygen release are the three primary techniques for research on photosynthesis. Measuring the chlorophyll fluorescence parameters of mutants helps to better understand the structural characteristics of their photosystems. The photosynthetic efficiency measured by chlorophyll fluorescence (Y[II] and ETR) was consistent with that measured by the LI-6400XT portable photosynthesis system. Moreover, Fo is independent of the photosynthetic light response but primarily depends on the structural state of the excitation energy that is transferred from the antenna pigment to the PSII (Krause and Weis, 1984). Disrupting or reversibly inactivating the PSII reaction center elevates Fo. Thus, the PSII of mutant could be abnormal. A combination of photosynthesis, chlorophyll fluorescence, and TEM indicated abnormal chloroplasts in the mutants.

A virescent leaf is a unique leaf color mutation with only one maternally inherited mutation identified in tobacco (Archer and Bonnett, 1987). Most virescent leaves are affected by temperature and light. However, the phenotype was only significant under high light intensity in bean (Dale and Heyes, 1970) and cotton (Mao et al., 2019). Most of the mutants were phenotypically significant under low temperature or low light. The rice *v1*, *v2*, and *v3* mutants (Iba et al., 1991), rice *virescent5a* (*osv5a*; Liu et al., 2016), maize *v16* (Edwards and Jenkins, 1988), maize *pale yellow-1* (Phinney and Kay, 1954), and wheat *virescent* (Gilmore and Tuleen, 1973) mutants are phenotypically significant at low temperatures. Tobacco *vir-c* (Archer and Bonnett, 1987) and cucumber *csvyl* (Song et al., 2018) have significant phenotypes under low light. Temperature-sensitive mutants are present in rice *virescent-albino leaf 1* (Zhang et al., 2018a) and *vyl* (Dong et al., 2013).

Under relatively low temperatures (21 and 28°C), the chlorophyll and carotenoid contents of the WT barely changed, and the pigment content of the mutant significantly increased. Temperature affected the mutant trans-color. At 35°C, the pigment content in the WT and the mutant increased simultaneously, probably because the high temperature increased the enzyme activity (Webb and Melis, 1995; Zhao, 2010). The pigment content of WT individuals was the highest under high temperatures at different periods and started to disintegrate on day 6. In contrast, the pigment content of the mutant did not degrade significantly on day 8, possibly because the high temperature accelerated leaf senescence. Under relatively low light (10,000 and 25,000 Lx), the mutant pigment content increased significantly, and the mutant was also sensitive to light. Under high light intensity (50,000 Lx) treatments, the pigment content of WT individuals did not increase significantly, but the chlorophyll content in the mutant decreased. Decreased chlorophyll contents have been observed several times due to high light intensity (Niinemets and Tenhunen, 1997; Ishida and Toma, 1999; Yulong et al., 2002; Zhao, 2010). These changes could be due to the poor stability of chlorophyll and photoinhibition caused by high light intensity, which causes photooxidation and destroys the chlorophyll (Anderson et al., 1997). The inconsistent responses of the WT and mutant to the same light intensity could be due to the significant difference in the light saturation point between the WT and the mutant. The mutant light saturation point was 12,700 Lx, while that of the WT was 20,700 Lx. Although the light intensity was set at 50,000 Lx, the light source was 55 cm above the plant, causing intensity attenuation.

Map-based cloning, the classical, well-established method for locating traits, was used to map the SNPs responsible for the WT and mutant traits. MutMap was first proposed in 2012 to localize mutagenic mutations (Abe et al., 2012). In this study, map-based cloning was combined with BSA re-sequencing. Thus, *v-3* was shown to be a single nuclear recessive mutation in the F_2_-A and F_2_-B populations. The *v-3* candidate gene maps between 33.5 and 35.7 Mb (chr3). Unfortunately, this position is a cold spot with no recombination events. Cold spots usually occur at the centromere regions, heterochromatin, and telomere positions (Wu et al., 2003; Si et al., 2015). In addition, traditional map-based cloning is very difficult at these locations. Some researchers have proposed various alternative methods, such as expanding the number and type of localized populations (Stein, 2009), BSR-Seq (Liu et al., 2012), mutagenesis (Morita et al., 2009; Buerstmayr et al., 2018), and gene editing (Liu et al., 2021a). However, these strategies require a substantial amount of time and effort and are subject to false positives. The MapRseq strategy successfully located a fragile gene in wheat (Deng et al., 2019). In this study, a combination of three strategies was used. The region 33.5–35.7 Mb of chr3 was localized by map-based cloning and narrowed by BSA re-sequencing. The DEGs in this region were identified from the transcriptome data, and the SNPs of DEGs were screened based on resequencing data. We identified only one nonsynonymous SNP in *RST1*. Thus, *RST1* was confirmed as the candidate gene for *v-3*.

The chloroplasts of higher plants originated 1–1.5 billion years ago *via* a symbiotic event within the cyanobacteria (Douzery et al., 2004; Yoon et al., 2004). Evolution has recently integrated most genes into the host cell nucleus (Timmis et al., 2004). The chloroplast has its genome. Thus, the chloroplast is a semi-autonomous organelle regulated by the nuclear and chloroplast genomes (McFadden, 2001). Chloroplast genes primarily encode the PEP core subunit, ribosomal proteins, chloroplast tRNA, and rRNA. Chloroplast genes are co-transcribed by PEP and NEP (Liu et al., 2021b). Transcriptome analysis revealed that the mutants did not significantly affect specific transcription, and the expression of chloroplast-related ribosomal genes was not affected. However, the expression of *RNA polymerase σ factor* and *plastid division protein*, PDV2, was significantly reduced. In addition, different reasons decreased the chlorophyll and carotenoid contents in the mutant. However, the expression of PEP-regulated genes was not reduced, excluding the assumption that the reduced expression of the *σ-factor* caused the phenotypic differences.

RNA exosomes, exoribonucleases, and multimeric cofactors, such as RNA helicases/Ski complex, poly(A) polymerases, and RST1, form a higher-order complex that catalyzes the maturation and degradation of all types of RNA (Lange and Gagliardi, 2022; Lee and Suh, 2022). Moreover, poly(A) polymerases catalyze the polyadenylation and exonucleolytic degradation of mRNA in chloroplasts (Yehudai-Resheff et al., 2001, 2003). The polymerase mutants *pnp1-1* and *rif10* in Arabidopsis have light green leaves (Sauret-Güeto et al., 2006; Marchive et al., 2009). The ski-like RNA helicase is required for RNA metabolism in chloroplasts. The Arabidopsis *ise2* mutant has white or light green leaves and abnormal embryonic development due to the ski-like RNA helicase mutation (Kobayashi et al., 2007). Silencing the *EMB175/PPR103* that interacts with the ski-like RNA helicase in tobacco results in yellow leaves (McCray, 2017). In addition, the *rst1* mutants have an abnormal leaf shape, late inflorescence emergence, and stunted embryo development, which results in nonviable seeds (Chen et al., 2005). In addition, RST1 connects the Ski complex to the RNA exosome (Lange et al., 2019; Li et al., 2019; Auth et al., 2021). Multiple virescent leaf mutants are caused by abnormalities in the basic functions of the chloroplast, including RNA metabolism (Kusumi et al., 2004; Sugimoto et al., 2007; Zhang et al., 2018b; Mao et al., 2019) and protein metabolism (Koussevitzky et al., 2007; Dong et al., 2013; Xing et al., 2014; Song et al., 2018).

This study was inspired by the regulation of *rst1* mutant in Arabidopsis (Daszkowska-Golec, 2020; Lee and Suh, 2022). A combination of RNA-Seq and TEM enabled this study to introduce a hypothesis that the *v-3* mutant causes leaf color mutations. In normal WT plants, the RNA degradation pathway (XRN4 and RNA exosome supercomplex) degrades excess PDV2 transcription products in the WT and then does not trigger post-transcriptional gene silencing (PTGS). However, in the *v-3* mutant, the RNA exosome function is abnormal, and the mRNAs of *PDV2* are already bound to the RNA exosome. They cannot be degraded by XRN4. Therefore, the accumulation of mRNA leads to PTGS, which ultimately determines the leaf color mutation.

## Data availability statement

The original contributions presented in the study are publicly available. This data can be found at: NCBI, PRJNA844414 and PRJNA843604.

## Author contributions

ML and SL designed the experiments. SL and GX provided experimental methods. ZZ performed the research and analyzed the data and wrote the manuscript. JW and SL reviewed the manuscript. All authors contributed to the article and approved the submitted version.

## Funding

This research was supported by the Innovative Talents Plan of Shanxi Agricultural University (BJRC201601), the Key Research and Development Project of Shanxi Province (201903D211011), and the Basic Research Project of Shanxi Province (Grant No. 20210302124368).

## Conflict of interest

The authors declare that the research was conducted in the absence of any commercial or financial relationships that could be construed as a potential conflict of interest.

## Publisher’s note

All claims expressed in this article are solely those of the authors and do not necessarily represent those of their affiliated organizations, or those of the publisher, the editors and the reviewers. Any product that may be evaluated in this article, or claim that may be made by its manufacturer, is not guaranteed or endorsed by the publisher.

## Supplementary material

The Supplementary Material for this article can be found online at: https://www.frontiersin.org/articles/10.3389/fpls.2022.972620/full#supplementary-material

## Footnotes

^1^ http://cucurbitgenomics.org/

^2^ www.megasoftware.net

^3^ http://linux1.softberry.com/berry.phtml
