# Supplementary material for: Integrating physiology, genetics, and transcriptome to decipher a new thermo-sensitive and light-sensitive virescent leaf gene mutant in cucumber
Source: Front Plant Sci. 2022 Aug 16;13:972620. doi: 10.3389/fpls.2022.972620 (PMC9424728; doi:10.3389/fpls.2022.972620)
Supplement: Supplementary file 1 [file Data_Sheet_1.docx]

**Supplementary Figure 1.** Chlorophyll b content of *v-3* and WT under different light intensity treatments .

**Supplementary Figure 2.** Chlorophyll b content of *v-3* and WT under different temperature treatments .


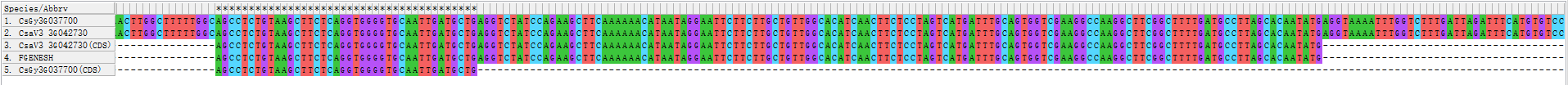


**Supplementary Figure 3.** The 13th exon sequence alignment of *RST1* gene in ‘9930’, ‘Gy14’, ‘The exons predicted by FGENESH’.


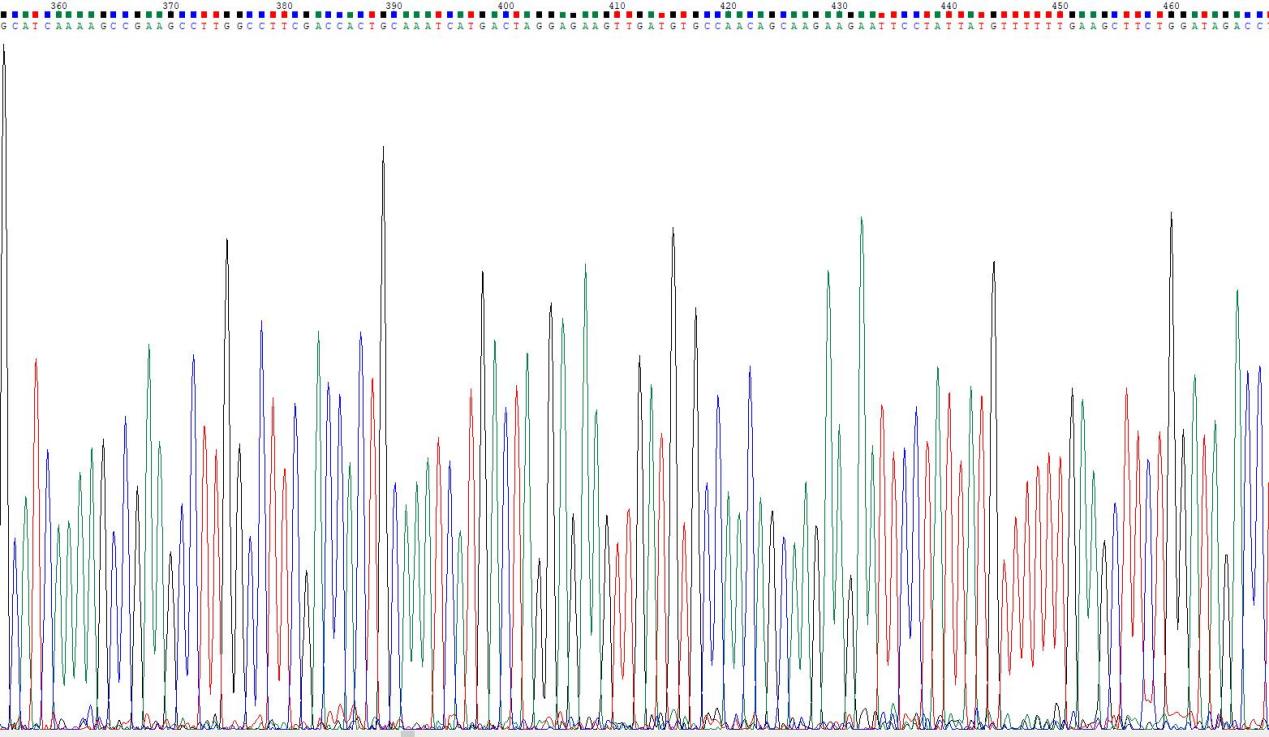


**Supplementary Figure 4.** Sequencing results of different parts of 13th exon of *RST1*.

**Supplementary Figure 5.** Verification of the expression of 9 genes obtained in RNA-seq by RT-qPCR.The value from the RT-PCR of *v-3*/SC311 (x-axis) was plotted against the RNA-seq Foldchange value(y-axis).


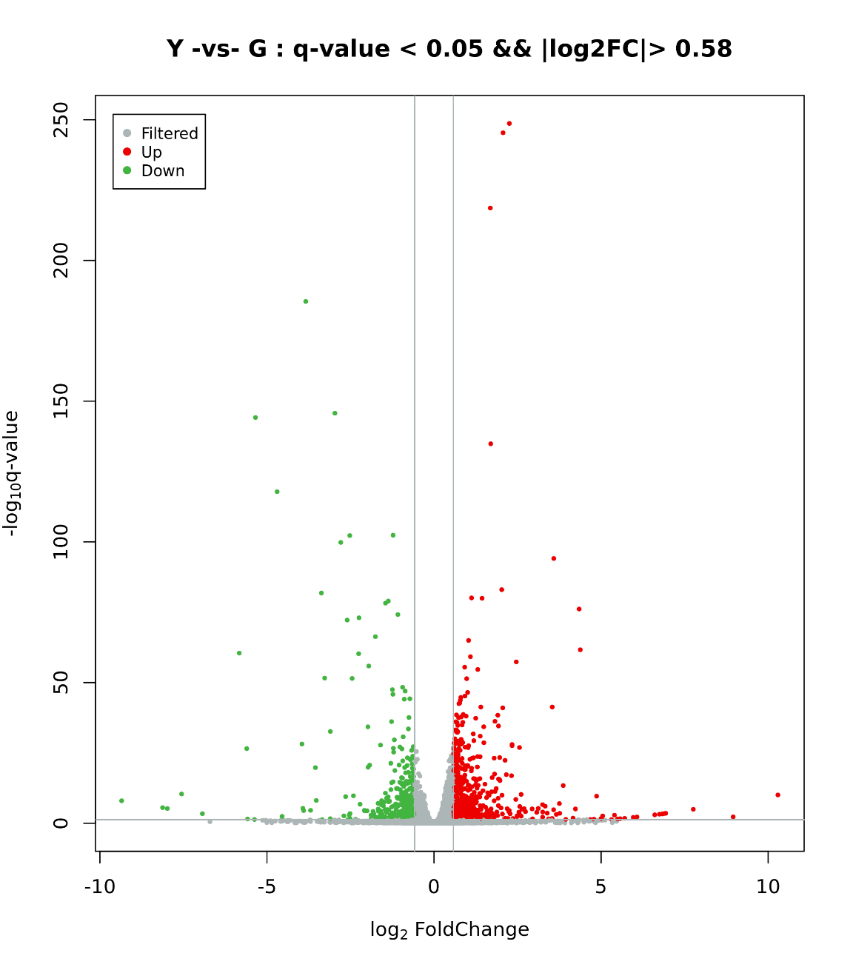


Contral: SC311

Case: *v-3*

UP: 643

DOWN: 461

NoDiff: 18204

**Supplementary Figure 6.**Volcano plot of upregulated and downregulated differentially

expressed genes (DEGs) in SC311 (WT) versus *v-3* (mutant). The log2 fold change (x-axis) is plotted against the log10 P-value (y-axis). Red dots represent DEGs that were upregulated upon SC311, whereas blue dots represent downregulated DEGs. Grey dots denote genes that were not significantly different between SC311 and *v-3* (mutant).


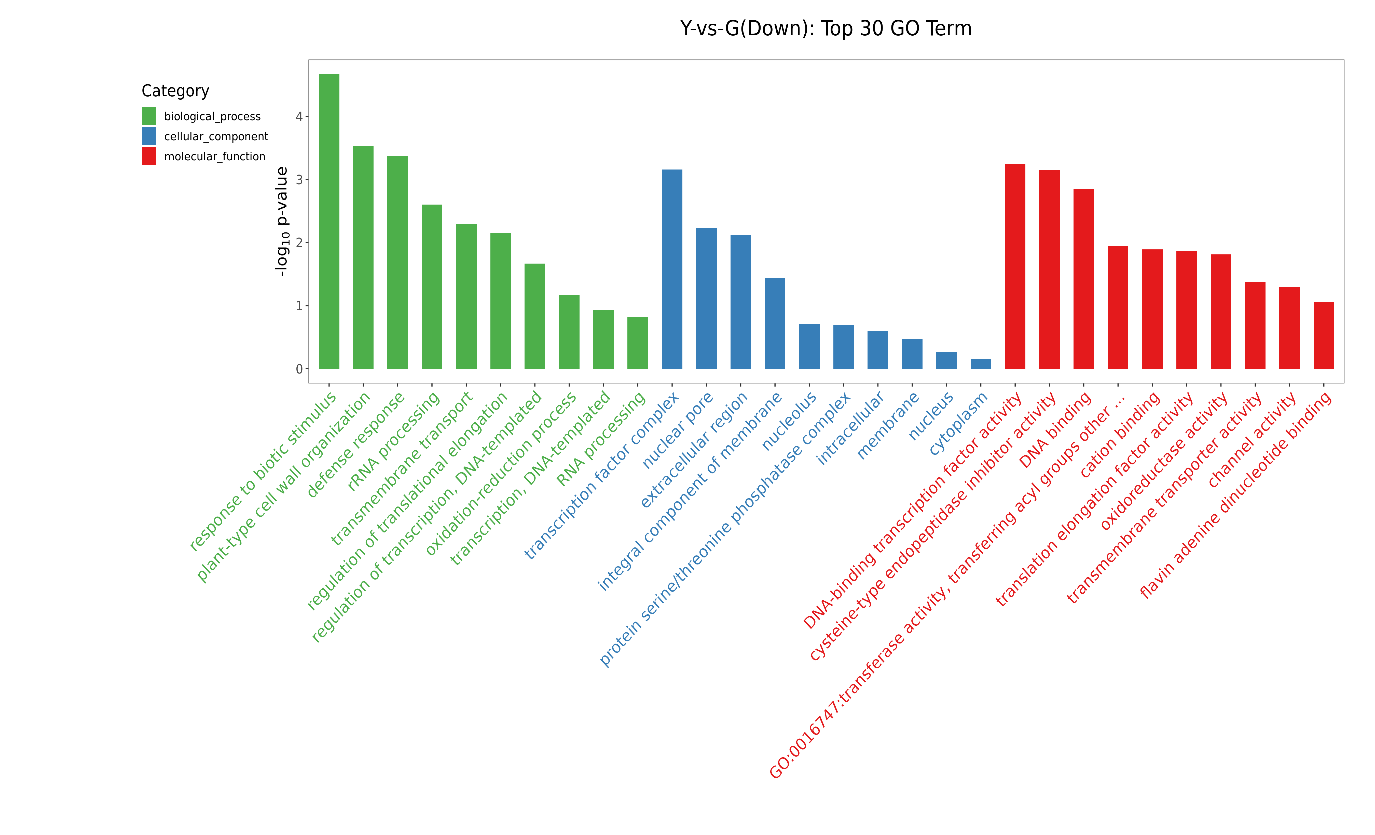


**Supplementary Figure 7.** Gene ontology (GO) annotation of differentially expressed genes (DEGs) in SC311 and *v-3* (mutant).


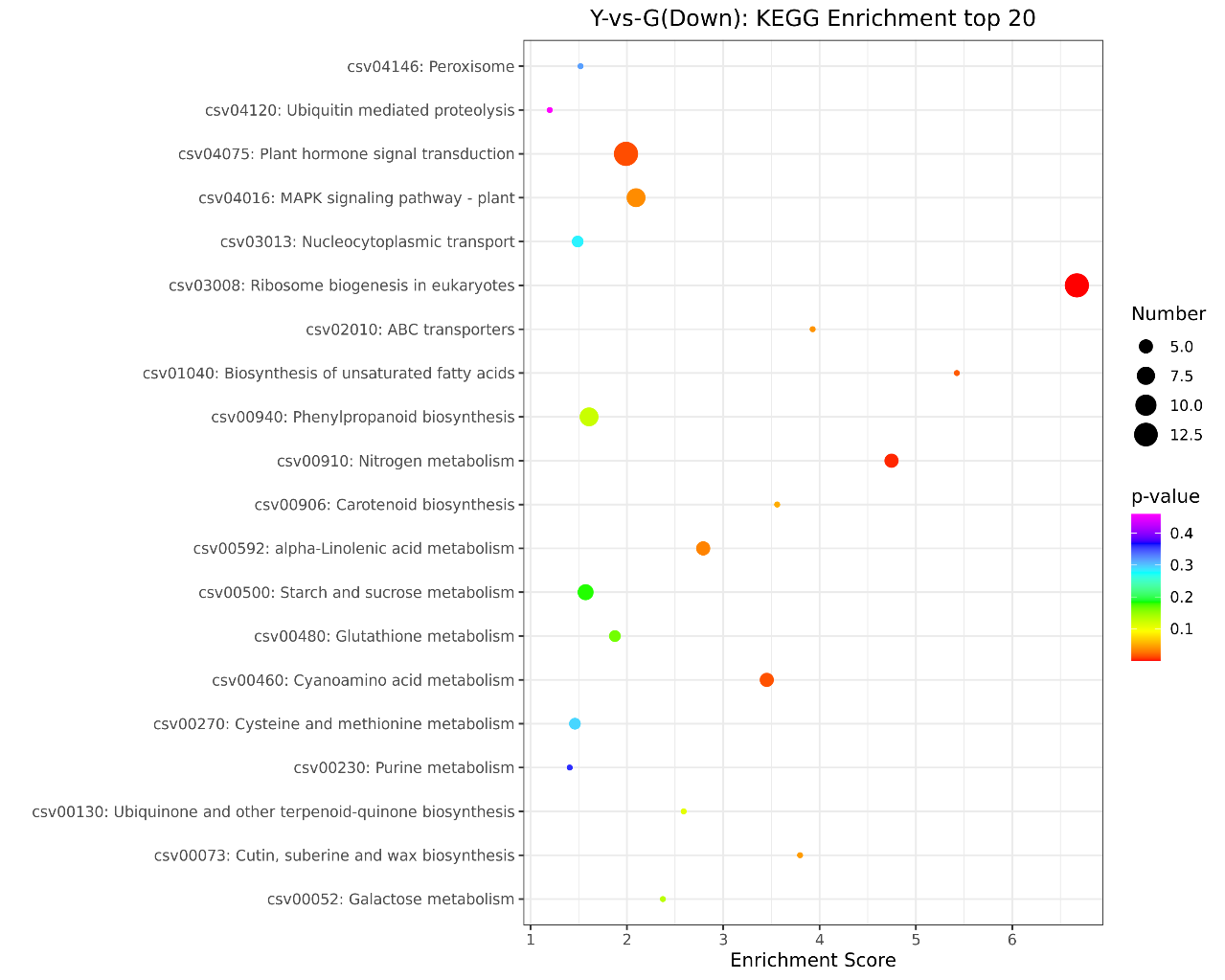


**Supplementary Figure 8.** Gene numbers significantly enriched KEGG pathways in up-regulated genes (Contral: SC311; Case: *v-3*).


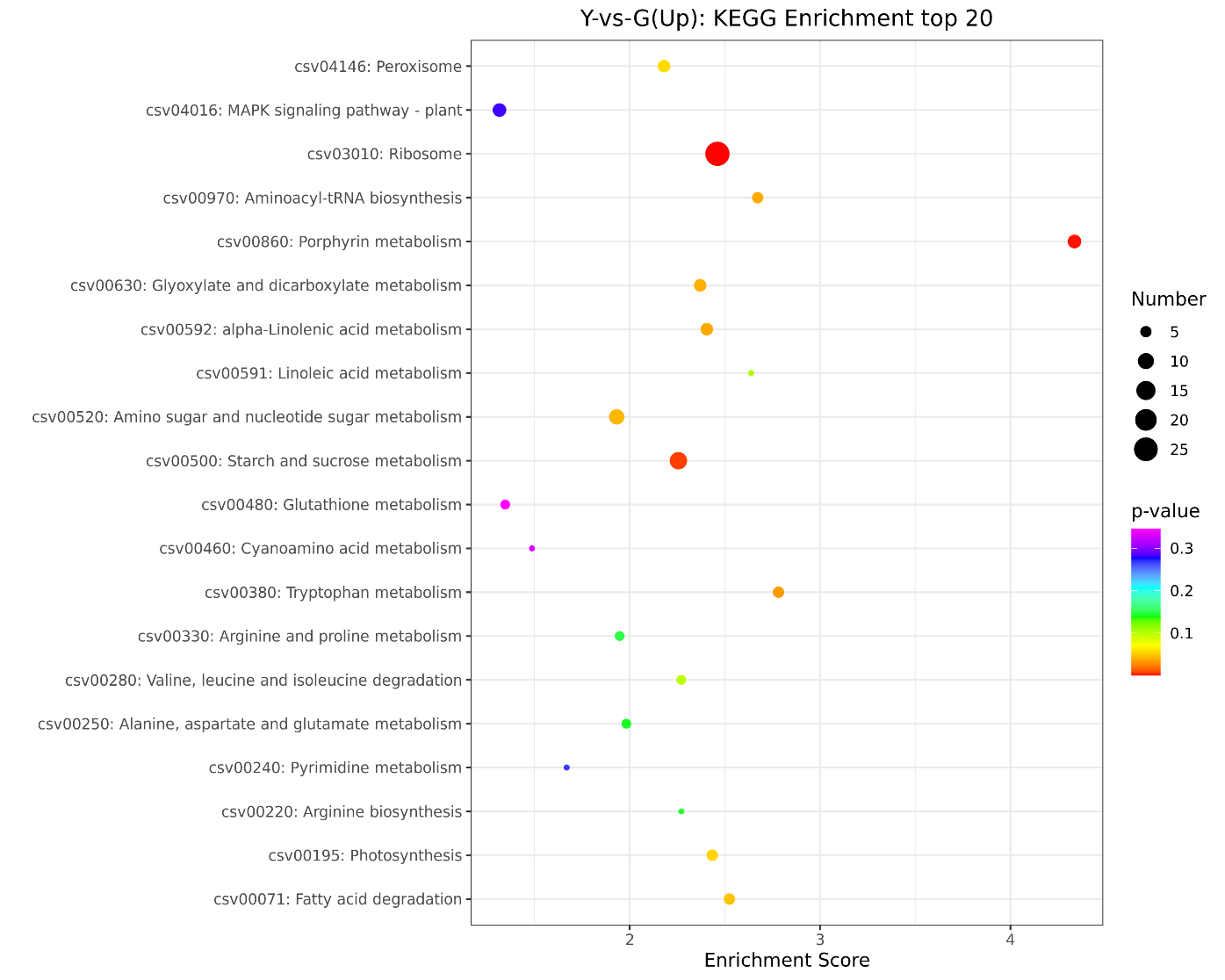


**Supplementary Figure 9.** Gene numbers significantly enriched KEGG pathways in down-regulated genes (Contral: SC311; Case: *v-3*).


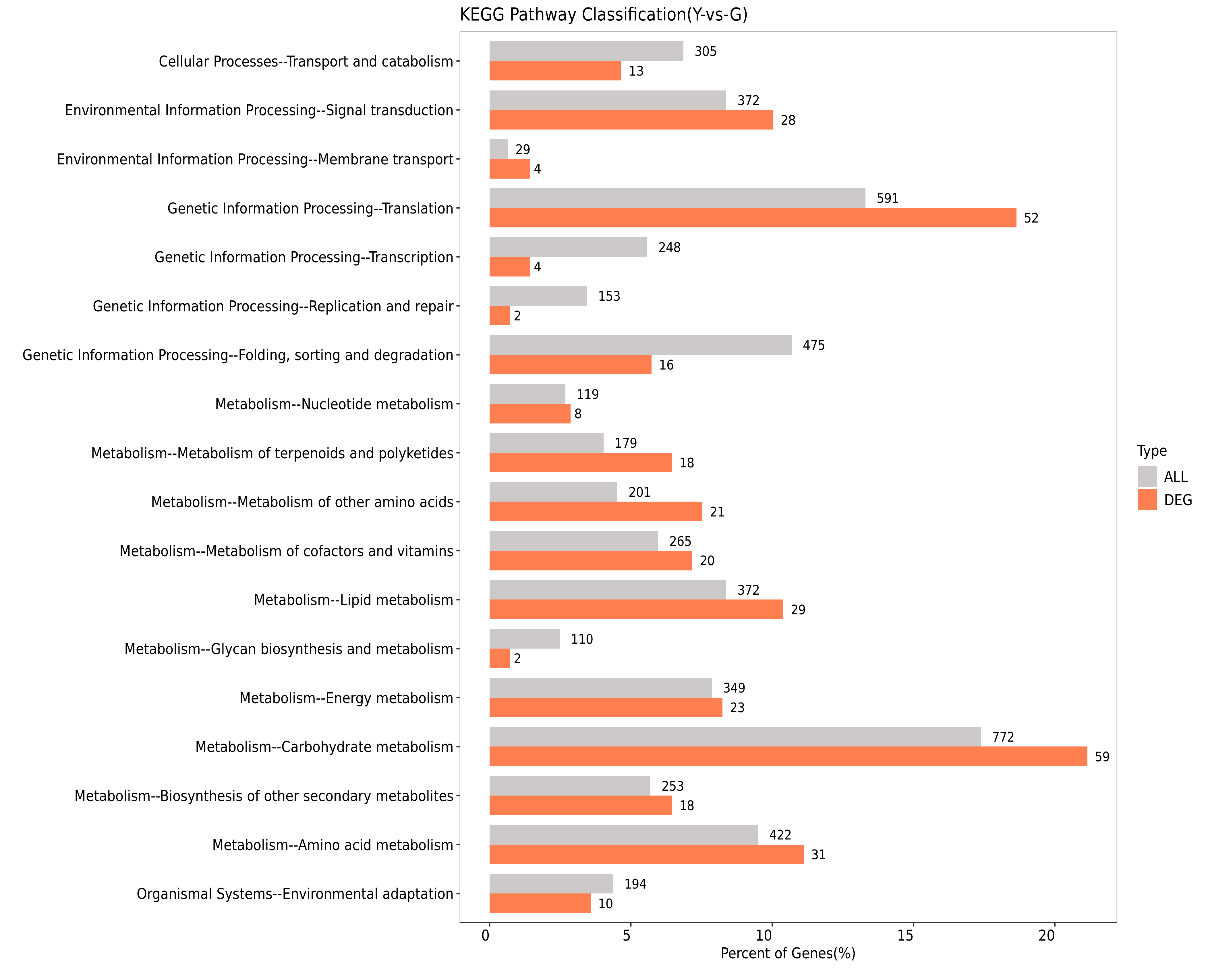


**Supplementary Figure 10.** Comparison of DEGs and all genes at KEGG pathways classification

**Supplementary Table 1.** Primers related to this study.

| Name | Left | Right | Notes |
| --- | --- | --- | --- |
| UW043643 | TCTCACACCCCATTTTCCTC | CCACCCCATTAGATATCCACC | Mapping |
| SSR20733 | AACCTCTCCATCCCCGATAC | TTCGAATGCTTGGATTTTCA | Mapping |
| SSR10783 | TGGGAAAATGGGAGTTTCAA | CGAACCACCAGTATTGGACC | Mapping |
| SSR18311 | GCGGATCAGAGAGGAAACAG | GAAACAAACGTCCTCCTCCA | Mapping |
| SSR15124 | TCCACCCAATCTTTCCACTC | AGTGCTCTTCACATTTGCCA | Mapping |
| UW084840 | ACACGTAGTCAGAAAAACATATAAA | TTAAACAAACCCAAATCTTTTCTTC | Mapping |
| UW083999 | TTTTCAACCTTTTCACTTTTGGA | CCACCCAACACCCACTTTTA | Mapping |
| UW71654 | TGTATGAGGATCACGATTGATTG | GGCATAGCCCAAAATTGCTA | Mapping |
| SSR29128 | CAAATGGCGTTTCACTTTCT | GGGTGAGTCCAAAAACAAAGA | Mapping |
| SSR06031 | TGGGAAGAGAACCCTAGGAAA | TTGCAATTACTCATCGCTGC | Mapping |
| UW084839 | TTACCAAGTTTGGAGGGGAAG | CAGAGTCGTCGAGTTGAAGTAGG | Mapping |
| SSR21456 | AAGTGGGAAGGTTTGGAGGT | TTCCTTTTTCCCCCTTCATT | Mapping |
| SSR04122 | GCCAGGATCAGAAGACGAAG | CTCTTCTCTCTCCCCGGACT | Mapping |
| UW070984 | ATCCATGAAAACGAAAGGAA | TGCACCATAGTTTCCGTGTT | Mapping |
| SSR07131 | TTCTCATGCTTCCTACCGCT | TTCTATTGGAGGGCTGGTTG | Mapping |
| CsRST1 | ATGGACTCGTATGCTCCATTTCTAGAGA | ACCAATTTCACGCATCGCATT | Clone |
| CsaV3_3G039990 | ACTCTTCTCTCCGAAACGACT | TGAATTCCAAGCCCTGTTCGTC | RT-qPCR |
| CsaV3_3G041700 | CATTGCCTACGGTGCTGGTCTTAG | GAGATGACATGGTGGATGCGAAGG | RT-qPCR |
| CsaV3_3G041740 | TGGTGTGGACCCTGTAGACT | CGGGCTCCAACCACTGTATC | RT-qPCR |
| CsaV3_3G042570 | TATAAAACTGATGGTATTGGGCTCT | CATCGGGAATGATAAGCAAC | RT-qPCR |
| CsaV3_3G042580 | GGTCGTAGAGGCAAGAGATGAGTTG | ATCCAAACCTTCGTACAGG | RT-qPCR |
| CsaV3_3G039870 | ATGAGAAGTTCCAGCGCAAA | TTCACTTGAACTGCGACCT | RT-qPCR |
| CsaV3_3G041030 | AACCTCGGCCTAAGATCCCT | CGCTCTCGTTAACACCAGGA | RT-qPCR |
| CsaV3_3G041110 | TTCGATTCACCTGCTAGGGC | AGCAGCACAGATTCACCAGT | RT-qPCR |
| CsaV3_3G041230 | AGACGATGTAGACAAAATAAGCCAA | TGACAATTCTACTACCTGAACCGAA | RT-qPCR |
| CsaV3_3G041690 | TGGTGATGAAAGTCGCCCTC | CTTCCTCAATGCCCGCACTA | RT-qPCR |
| action | CTCCACTCAACCCAAAGGCTA | AGAATCCAGCACGATACCAGT | RT-qPCR |

**Supplementary Table 2.** Mapped region of *v-3* locus by BSA-Seq.

| Chromosome ID | Start | End | Size(Mb) |
| --- | --- | --- | --- |
| Chr3 | 34660000 | 34710000 | 0.05 |
| Chr3 | 34900000 | 35580000 | 0.68 |
| Chr3 | 35600000 | 37350000 | 1.75 |
| Chr3 | 37470000 | 37530000 | 0.06 |
| Total |  | | 2.54 |

**Supplementary Table 3.** The location region of *v-3.*

| Chromosome ID | Start | End | Size(Mb) |
| --- | --- | --- | --- |
| Chr3 | 34660000 | 34710000 | 0.050001 |
| Chr3 | 34900000 | 35580000 | 0.680001 |
| Chr3 | 35600000 | 35661144 | 0.61144 |
|  |  |  | 1.341442 |

**Supplementary Table 4.** Distribution of missense SNPs eligible for screening in the location region.

| Position | Ref | *v-3* | Gene | Amino acid | Annotation |
| --- | --- | --- | --- | --- | --- |
| 33698102 | G | C | CsGy3G035130 | Met/Ile | Cytochrome P450, putative |
| 33710463 | A | G | CsGy3G035150 | Thr/Ala | Protein kinase |
| 33712306 | A | G |  | Ser/Gly |  |
| 33712385 | G | A |  | Arg/Gln |  |
| 33713659 | T | C |  | Val/Ala |  |
| 34862820 | C | G | CsGy3G036830 | Val/Leu | Pectate lyase |
| 35316742 | T | A | CsGy3G037450 | Gln/Leu | Uncharacterized protein |
| 35357378 | A | G | CsGy3G037510 | Ser/Pro | Unknown Protein |
| 35357491 | C | A |  | Ser/Ile |  |
| 35357497 | A | G |  | Val/Ala |  |
| 35357519 | T | C |  | Lys/Glu |  |
| 35357525 | C | G |  | Val/Leu |  |
| 35366128 | T | C | CsGy3G037530 | Ile/Val | Unknown Protein |
| 35392452 | C | T | CsGy3G037560 | His/Tyr | Peptide deformylase |
| 35393696 | T | C |  | Ser/Pro |  |
| 35405309 | C | T | CsGy3G037580 | Met/Ile | Polygalacturonase |
| 35405334 | C | T |  | Ser/Asn |  |
| 35405697 | A | G |  | Trp/Arg |  |
| 35496653 | A | G | CsGy3G037660 | Asn/Asp | NAC domain-containing protein 83-like |
| 35496662 | G | A |  | Asp/Asn |  |
| 35504973 | A | G | CsGy3G037690 | Asn/Ser | zinc finger CCCH domain-containing protein 41 isoform X1 |
| 35505097 | G | T |  | Glu/Asp |  |
| 34677616 | C | G | CsaV3_3G042730 | Ser/Thr | protein RST1 isoform X1 |

**Supplementary Table 5.** All differentially expressed genes in the candidate interval.

| gene_id | gene_id | FoldChange | q-value | description |
| --- | --- | --- | --- | --- |
| CsaV3_3G039930 | CsGy3G035090 | 1.559799122 | 4.80E-21 | protein NRT1/ PTR FAMILY 3.1-like |
| CsaV3_3G039970 | CsGy3G035120 | 4.990727625 | 1.25E-17 | WAT1-related protein |
| CsaV3_3G040080 | CsGy3G035210 | 1.700054089 | 0.013256487 | Pentatricopeptide repeat-containing protein, mitochondrial |
| CsaV3_3G040160 | - | 0.312134068 | 0.007310661 | sigma factor binding protein 2, chloroplastic |
| CsaV3_3G040200 | CsGy3G035310 | 4.56079936 | 6.48E-06 | Amino acid transporter family protein |
| CsaV3_3G040210 | CsGy3G035310 | 2.91181343 | 1.86E-06 | Amino acid transporter |
| CsaV3_3G040580 | CsGy3G035630 | 2.485523984 | 0.000184686 | Beta-glucosidase |
| CsaV3_3G040670 | CsGy3G035740 | 0.602878534 | 0.013887238 | RRP12-like protein |
| CsaV3_3G040740 | CsGy3G035880 | 0.651729473 | 0.000119025 | Nuclear pore protein |
| CsaV3_3G040820 | CsGy3G035950 | 1.656273031 | 2.47E-08 | EF hand calcium-binding family protein |
| CsaV3_3G041130 | CsGy3G036250 | 0.509538447 | 0.013580152 | pentatricopeptide repeat-containing protein At1g61870, mitochondrial-like |
| CsaV3_3G041150 | CsGy3G036270 | 0.613403235 | 2.59E-23 | Cystathionine gamma synthase |
| CsaV3_3G041460 | CsGy3G036580 | 0.641786582 | 1.13E-08 | aquaporin NIP2-1-like |
| CsaV3_3G041510 | CsGy3G036650 | 0.512991985 | 0.000369707 | myb-related protein 306-like |
| CsaV3_3G041560 | CsGy3G036690 | 0.175679334 | 0.024298024 | Integrin beta-like protein |
| CsaV3_3G041890 | - | 216.8313782 | 1.10E-05 | Unknown protein |
| CsaV3_3G041910 | CsGy3G037010 | 0.401465989 | 0.001192909 | Cyclic nucleotide-gated ion channel, putative |
| CsaV3_3G042050 | CsGy3G037160 | 1.647684128 | 8.10E-05 | senescence-associated carboxylesterase 101 |
| CsaV3_3G042070 | CsGy3G037180 | 1.542143101 | 1.03E-16 | EG2771 |
| CsaV3_3G042150 | CsGy3G037220 | 0.467480837 | 4.14E-13 | WAT1-related protein |
| CsaV3_3G042160 | CsGy3G037220 | 0.279715469 | 0.03846067 | WAT1-related protein |
| CsaV3_3G042400 | CsGy3G037400 | 1.554984476 | 0.028327365 | Deoxynucleoside triphosphate triphosphohydrolase samhd1 like |
| CsaV3_3G042410 | CsGy3G037410 | 1.568190334 | 1.59E-17 | Calcium homeostasis regulator CHoR1, putative |
| CsaV3_3G042510 | CsGy3G037500 | 1.70471965 | 1.83E-43 | 50S ribosomal protein L7/L12 |
| CsaV3_3G042660 | CsGy3G037630 | 0.65861895 | 0.000594546 | cysteine-rich receptor-like protein kinase 42 |
| CsaV3_3G042690 | CsGy3G037650 | 0.472564331 | 6.76E-75 | Raffinose synthase |
| CsaV3_3G042730 | CsGy3G037700 | 0.647460581 | 0.004476998 | protein RST1 isoform X1 |
| CsaV3_3G042760 | CsGy3G037730 | 52.21054471 | 0.017498148 | LOB domain-containing protein, putative |
| CsaV3_3G042980 | - | 0.371513771 | 0.000119667 | Unknown protein |

**Supplementary Table 6.** Genes involved in chlorophyll and carotenoid biosynthesis and decomposition pathways.

| Gene name | Description | Gene_id |
| --- | --- | --- |
| GluTS | glutamyl-tRNA synthetase | CsaV3_7G002150 |
|  |  | CsaV3_1G038230 |
| GluTR | Glutamyl-tRNA reductase | CsaV3_2G030530 |
| GSA-AM | Glutamate-1-semialdehyde-2,1-aminomutase | CsaV3_7G007260 |
| ALAD | δ-Aminolevulinic acid dehydratase | CsaV3_2G033150 |
| PBGD | Porphobilinogen deaminase | CsaV3_3G031800 |
| UROS | Uroporphyrinogen Ⅲ synthase | CsaV3_2G028140 |
|  |  | CsaV3_1G028580 |
| UROD | Uroporphyrinogen Ⅲdecarboxylase | CsaV3_5G010770 |
|  |  | CsaV3_4G008050 |
| CPOX | Coproporphyrinogen Ⅲoxidase | CsaV3_1G000810 |
| PPOX | Protoporphyrinogen Ⅸ oxidase | CsaV3_6G001080 |
|  |  | CsaV3_5G018640 |
| CHLH | Magnesium chelatase H subunit | CsaV3_4G011730 |
| MgPMT | Mg-protoporphyrin Ⅸ methyltransferase | CsaV3_1G000080 |
| MgPMEC | Mg-Proto Ⅸ monomethylester cyclase | CsaV3_4G031720 |
| DVR | 3,8-divinyl Chlide 8-vinyl reductase | CsaV3_3G014130 |
| POR | NADPH：Protochlorophyllide oxidoreductase | CsaV3_4G033870 |
| CAO | Chlorophyllide a oxygenase | CsaV3_6G034450 |
| NYC1 | chlorophyll(Ide) b reductase NOL, chloroplastic isoform X1 | CsaV3_6G004470 |
|  |  | CsaV3_1G024260 |
| HCAR | 7-hydroxymethyl chlorophyll a reductase, chloroplastic | CsaV3_3G011480 |
| CLHs | chlorophyllase-2, chloroplastic | CsaV3_2G013440 |
|  | chlorophyllase-1-like | CsaV3_5G025230 |
|  |  | CsaV3_2G013640 |
| MCS | magnesium dechelatase SGRL, chloroplastic | CsaV3_5G003370 |
|  |  | CsaV3_6G031220 |
| PAO | pheophorbide a oxygenase, chloroplastic | CsaV3_1G014120 |
| RCCR | red chlorophyll catabolite reductase | CsaV3_2G016720 |
|  |  | CsaV3_2G016730 |
| PSY | Phytoene synthase | CsaV3_5G020340 |
|  |  | CsaV3_4G023380 |
|  |  | CsaV3_4G007560 |
| PDS | Phytoene desaturase | CsaV3_4G002690 |
|  |  | CsaV3_1G045650 |
| ZDS | ζ-carotene desaturase | CsaV3_1G045650 |
| ε-LCY/LCYE | Lycopene ε-cyclase | CsaV3_2G001370 |
| β-LCY/LCYB | Lycopene-β-cyclase | CsaV3_4G022080 |
|  |  | CsaV3_4G000740 |
| LUT1 | Carotenoid ε-hydroxylase | CsaV3_6G040180 |
| CrtZ/BCH | β-carotene 3-hydroxylase | CsaV3_5G002530 |
|  |  | CsaV3_5G002520 |
|  |  | CsaV3_3G017450 |
| LUT5 | β-cyclohydroxylase | CsaV3_3G007050 |
| ZEP | Zeathanxin epoxidase | CsaV3_2G016630 |
| VDE | Violaxanthin deepoxidase | CsaV3_2G010250 |

**Supplementary Table 7.** PEP and NEP transcribes genes.

| Type | Gene name | description | gene_id |
| --- | --- | --- | --- |
| ClassⅠ | psaA | photosystem I P700 chlorophyll a apoprotein A1 | CsaV3_UNG203680 |
|  |  |  | CsaV3_UNG201530 |
|  |  |  | CsaV3_UNG203670 |
|  | psbA | photosystem II protein D1 | CsaV3_1G025480 |
|  |  |  | CsaV3_UNG208380 |
|  | petb | cytochrome b6 | CsaV3_4G007360 |
|  | rbcL | Ribulose bisphosphate carboxylase large chain | CsaV3_UNG203690 |
|  | ndhA | NADH dehydrogenase | CsaV3_UNG208120 |
|  |  |  | CsaV3_UNG208130 |
|  |  |  | CsaV3_6G024190 |
|  |  |  | CsaV3_6G025230 |
|  |  |  | CsaV3_6G025260 |
|  |  |  | CsaV3_6G025300 |
|  |  |  | CsaV3_UNG208480 |
|  |  |  | CsaV3_UNG208520 |
|  |  |  | CsaV3_UNG208530 |
|  |  |  | CsaV3_UNG208550 |
|  |  |  | CsaV3_UNG210330 |
|  |  |  | CsaV3_4G030790 |
| ClassⅡ | atpl | V-type proton ATPase proteolipid subunit | CsaV3_3G010990 |
|  |  |  | CsaV3_2G029310 |
|  |  |  | CsaV3_2G029320 |
|  | rps16 | ribosomal protein S16 | CsaV3_1G040590 |
|  |  |  | CsaV3_3G018380 |
|  |  |  | CsaV3_7G008490 |
|  |  |  | CsaV3_3G018990 |
|  | clpp | clp protease proteolytic subunit | CsaV3_2G006650 |
|  |  |  | CsaV3_2G014590 |
|  |  |  | CsaV3_3G001040 |
|  |  |  | CsaV3_3G002300 |
|  |  |  | CsaV3_3G021700 |
|  |  |  | CsaV3_3G031380 |
|  |  |  | CsaV3_4G001270 |
|  |  |  | CsaV3_5G003660 |
|  |  |  | CsaV3_7G007880 |
|  |  |  | CsaV3_4G026170 |
|  | ycf1 | Protein TIC 214 | CsaV3_1G025490 |
|  |  |  | CsaV3_1G025500 |
|  |  |  | CsaV3_2G013000 |
| ClassⅢ | rpoA | RNA polymerase subunit alpha | CsaV3_3G019080 |
|  | rpoB | RNA polymerase subunit beta | CsaV3_UNG208340 |
|  |  |  | CsaV3_UNG208330 |
|  |  |  | CsaV3_6G035750 |
|  |  |  | CsaV3_3G039630 |
|  |  |  | CsaV3_6G035730 |
|  |  |  | CsaV3_7G035430 |
|  |  |  | CsaV3_UNG208350 |
|  |  |  | CsaV3_3G037910 |
|  |  |  | CsaV3_6G007910 |
|  |  |  | CsaV3_4G036560 |
|  |  |  | CsaV3_6G045590 |
|  |  |  | CsaV3_3G007920 |
|  |  |  | CsaV3_1G010810 |
|  |  |  | CsaV3_3G046600 |
|  |  |  | CsaV3_6G050130 |
|  |  |  | CsaV3_3G023810 |
|  |  |  | CsaV3_1G029210 |
|  |  |  | CsaV3_3G038620 |
|  | rpoC1 | RNA polymerase subunit beta' | CsaV3_UNG208350 |
|  | rpoC2 | DNA-directed RNA polymerase subunit beta'' | CsaV3_UNG208360 |
|  |  |  | CsaV3_UNG207810 |
|  |  |  | CsaV3_5G013200 |
|  | ycf2 | Protein Ycf2 | CsaV3_UNG209000 |

**Supplementary Table 8.** Chloroplast ribosomal protein gene.

| Gene name | description | gene_id |
| --- | --- | --- |
| RP3 | 30S ribosomal protein 3, Chloroplastic-like | CsaV3_3G020570 |
|  |  | CsaV3_5G023320 |
|  |  | CsaV3_UNG207820 |
| RPS3 | 30S ribosomal protein S3, Chloroplastic | CsaV3_4G010600 |
|  |  | CsaV3_4G026180 |
| RPS36 | 30S ribosomal protein S6 alpha, Chloroplastic | CsaV3_5G001930 |
| RPS7 | 30S ribosomal protein S7, Chloroplastic | CsaV3_UNG208960 |
| RP5 | 50S ribosomal protein 5, Chloroplastic | CsaV3_4G029150 |
| RPL13 | 50S ribosomal protein L13 Chloroplastic | CsaV3_3G044830 |
| RPL19 | 50S ribosomal protein L19, Chloroplastic | CsaV3_3G002360 |
| RPL2 | 50S ribosomal protein L2, Chloroplastic | CsaV3_2G006410 |
| RPL2 | 50S ribosomal protein L2, Chloroplastic | CsaV3_UNG209010 |
| RPL29 | 50S ribosomal protein L29, Chloroplastic | CsaV3_4G008900 |
| RPL34 | 50S ribosomal protein L34, Chloroplastic | CsaV3_6G050830 |
| RPS18 | 30S ribosomal protein S18, Chloroplastic | CsaV3_7G024620 |

**Supplementary Table 9.** RNA polymerase sigma factor and plastid division protein gene.

| Gene name | description | gene_id |
| --- | --- | --- |
| SIG | RNA polymerase sigma factor | CsaV3_3G021990 |
|  | RNA polymerase sigma factor | CsaV3_2G035830 |
|  | RNA polymerase sigma factor | CsaV3_6G006360 |
|  | RNA polymerase sigma factor sigC | CsaV3_2G030610 |
| PDV2 | plastid division protein PDV2 | CsaV3_2G014470 |
| PDV2 | plastid division protein PDV2 | CsaV3_7G010190 |
| PDV1 | plastid division protein PDV1 | CsaV3_3G021850 |
